# Recurrent SARS-CoV-2 Omicron broadly neutralizing humanized antibodies in different single human V_H_1-2-rearranging mouse models

**DOI:** 10.1073/pnas.2537053123

**Published:** 2026-03-23

**Authors:** Himanshu Batra, Sai Luo, Kevin O. Saunders, Jaclyn S. Higgins, Fanchong Jian, Jun Zhang, Md Golam Kibria, G. M. Jonaid, Qingchen J. Zhou, Amanda Eaton, Kenneth Cronin, Michael L. Mallory, Melissa Mattocks, Robert J. Edwards, Robert Parks, Esther M. Lee, Adam Yongxin Ye, Aimee Chapdelaine Williams, Geeyoun Jung, Katayoun Mansouri, S. Munir Alam, David C. Montefiori, Ming Tian, Ralph S. Baric, Yunlong Cao, Barton F. Haynes, Bing Chen, Frederick W. Alt

**Affiliations:** ^a^Howard Hughes Medical Institute, Boston Children’s Hospital, Boston, MA 02115; ^b^Program in Cellular and Molecular Medicine, Boston Children’s Hospital, Boston, MA 02115; ^c^Department of Genetics, Harvard Medical School, Boston, MA 02115; ^d^Duke Human Vaccine Institute, Duke University School of Medicine, Durham, NC 27710; ^e^Department of Surgery, Duke University, Durham, NC 27710; ^f^Department of Integrative Immunobiology, Duke University School of Medicine, Durham, NC 27710; ^g^Departments of Epidemiology and Microbiology and Immunology, University of North Carolina at Chapel Hill, Chapel Hill, NC 27599; ^h^Biomedical Pioneering Innovation Center, Peking University, Beijing 100871, China; ^i^Changping Laboratory, Beijing 102206, China; ^j^Division of Molecular Medicine, Boston Children’s Hospital, Harvard Medical School, Boston, MA 02115; ^k^Department of Pediatrics, Harvard Medical School, Boston, MA 02115; ^l^Department of Medicine, Duke University School of Medicine, Durham, NC 27710; ^m^Department of Immunology, Duke University School of Medicine, Durham, NC 27710

**Keywords:** antibodies, V(D)J recombination, humanized mouse model, CDR3 diversity, SARS-CoV-2

## Abstract

During V(D)J recombination, antibody diversity is enhanced by nontemplated junctional modifications that generate immensely diverse heavy chain (HC) and light chain (LC) complementarity-determining 3 antigen-contact regions (CDR3s). We previously developed a mouse model that generates diverse antibody repertoires by rearranging a single human V_H_1-2 and Vκ1-33, associated with highly diverse CDR3s generated by V(D)J recombination with mouse Ds and/or Js. Immunization of this model with SARS-CoV-2 D614G spike elicited an antibody that potently neutralized SARS-CoV-2 variants through Omicron BA.2.754. Here, we report a related mouse model in which a single V_H_1-2 rearranges to human D3-3 and J_H_6, generating diverse HC-CDR3s much longer on average than those of our prior model. Omicron BA.4/.5 spike-ferritin nanoparticle-immunization of the new model elicited four highly related humanized antibodies that potently neutralize downstream Omicron subvariants. All four antibodies had 12 AA HC-CDR3s with two aromatic amino acids that engage an epitope comprising a hydrophobic patch opened-up by early Omicron lineage mutations and conserved in subsequent variants. Immunization of our prior, shorter CDR3-based model, elicited slightly less potent neutralizing antibodies that bound the same Omicron epitope, and were similar in all other aspects to those from the long, fully human CDR3 model. One tested antibody from each set reduced lung viral titers in a mouse-adapted BQ1.1 challenge. The antibodies we describe are related in their epitope recognition to recently described antibodies from Omicron-infected humans. These studies validate the utility of single human V_H_- and Vκ-rearranging mice for discovering humanized antibodies that neutralize emerging pathogens.

## Significance

Mouse models that generate antibody repertoires from human gene segments that predominantly encode single antibody lineages are useful for evaluating preclinical HIV-1 vaccine strategies. One such model was used for discovery of a humanized antibody that potently neutralized SARS-CoV-2 variants through the first several Omicron variants. Here, we describe a new, related humanized mouse model that generates a different antibody repertoire. Immunization with a SARS-CoV-2 Omicron spike protein immunogen elicited related humanized antibodies from both models that potently neutralized Omicron variants, with antibodies from the new model being more potent. Both sets of new antibodies reduced Omicron BQ1.1 viral titers in mouse models. These studies validate use of such mouse models for discovery of humanized antibodies against newly evolving pathogens.

Immunoglobulin (*Ig*) heavy chains (HCs) and light chains (LCs) are the basic subunits of the B cell receptor (“BCR”) and antibodies. In progenitor B cells, V(D)J recombination assembles exons that encode the HC and LC variable regions from component gene segments (1, 2). In mice, Ig HC variable region exons are assembled from approximately 100 V_H_, 10 D, and four J_H_ gene segments organized in a cluster from 5‘- to 3’ across the upstream end of the Ig HC locus (“*Igh”*) (1). *Ig* Kappa(κ) and *Ig* lambda(λ) LC variable region exons are assembled in precursor B cells from just V and J gene segments (3). In mice, most expressed Ig LCs are from the Ig kappa locus (*Ig*κ*),* which contains approximately 100 Vκs upstream of four Jκs (4). *Ig*κ is similarly organized in mice and humans, but human Igλ is more complex than that of mice and encodes a higher fraction of the human Ig LC repertoire (3). The antigen recognition portion of a given BCR or antibody is generated through interaction of 3 distinct sequence regions in each HC or LC variable region, collectively termed complementarity-determining regions (CDRs). Two CDRs (CDR1 and CDR2) are encoded by each V_H_ and V_L_ gene segment, with their sequence often unique to a particular V_H_ or V_L_ (5). In contrast, CDR3-encoding sequences of given HC or LC variable regions are, in significant part, generated, respectively, by V_H_(D)J_H_ and V_L_J_L_ junctional sequences (6). In this regard, V(D)J junctional sequences are diversified during the V(D)J recombination joining phase, which employs the nonhomologous DNA end-joining double-stranded break repair pathway (1). Such junctional diversification processes include deletion of potential coding sequences during end-processing and insertions of nontemplated nucleotides by terminal deoxynucleotidyl transferase (TdT) (2). While assortment of different V_H_ and V_L_ sequences, each often having unique CDR1 and 2 sequences, leads to hundreds of thousands of different antibodies, the huge number of different junctional-diversified CDR3-encoding sequences that can be associated with individual V_H_s and, to a lesser extent, V_L_s provide many orders of magnitude more unique variable region exon sequences than V(D)J recombination-based V_H_ and V_L_ assortment per se (7, 8).

Due to V(D)J recombination junctional diversification, the vast majority of newly generated B cells individually express a B cell receptor composed of a uniquely generated set of IgH and IgL variable region exons that usually differ distinctly from each other with respect to their HC and LCCDR3s (1, 2). Exceptions to this generalization occur in fetal B cell development where absence of TdT expression results in generation of recurrent microhomology-mediated “canonical” V(D)J CDR3s, and in mouse precursor B cells that, unlike those of humans, do not express TdT and, thus, express a less diverse LC-CDR3 repertoire than that of humans (9, 10). Still, in both mice and humans, the potential number of unique CDR3 sequences that can be generated in the adult BM has been estimated to exceed the number of newly generated B cells by many orders of magnitude, particularly for HC-CDR3 sequences (7, 8). In this regard, the size of the primary BCR repertoire expressed in a given mouse versus human immune system is determined, in large part, by the primary (newly generated) B cell numbers in their immune systems (11). Once generated in the BM, B cells move to the periphery where, upon activation by cognate antigens, they can be recruited to germinal centers (GCs) and undergo a somatic hypermutation (SHM) process that diversifies sequences of all three HC and LC-CDRs. Subsequently, the GC-specific affinity maturation process selects for B cells that express higher-affinity BCRs and have the potential to produce higher-affinity antibodies (12).

People living with HIV can develop neutralizing antibodies against viral coat protein epitopes that are conserved among diverse HIV-1 strains. These HIV-1 broadly neutralizing antibodies (“bnAbs”) evolve with HIV-1 virus over long infection periods. Developing vaccination approaches to elicit HIV-1 bnAbs is a major goal of the HIV-1 vaccine field. One challenge in eliciting HIV-1 bnAbs is that they often arise from precursor B cells that infrequently exist in the human population. Another challenge is that the mature bnAbs often have substantial numbers of SHMs, suggesting that they experienced many rounds of SHM in GCs during coevolution with the virus (13). Due to such limitations, testing approaches are frequently done in animal models (14). VRC01-class bnAbs target the conserved CD4-binding site epitope on the HIV-1 envelope and exclusively use human IGHV1-2*02 (termed V_H_1-2 from here forward), as the germline V_H_1-2 CDR2 encodes the majority of the interface of VRC01 bnAbs with the HIV-1 envelope (15). The IgL chains of VRC01 antibodies are frequently encoded by one of several different Vκs, including Vκ1-33 (16). Various humanized mouse models have been used to test the preclinical efficacy of candidate VRC01 lineage immunogens (17). We developed a mouse model for testing VRC01 class immunogens based on a strategy that allows V_H_1-2 and Vκ1-33 variable region exons to be developmentally assembled in association with diverse CDR3s via V(D)J recombination (18). This mouse model ectopically expresses a transgene allele that encodes human TdT, which substantially increases the diversity of Vκ1-33-based variable region exons CDR3s. We referred to this mouse model as a “complete” VRC01-rearranging model, with its key feature being generation of individual B cells that express many different potential VRC01-class precursor BCRs, as would be expected to occur in human VRC01 precursor repertoires (18). In this regard, such a model, can generate a much larger number of VRC01-class bnAb precursors than are generated in standard Immunoglobulin *Ig* gene–humanized mice, that rearrange the full complement of human *Igh* and *IgL* variable region gene segments in the context of a nearly 1,000-fold smaller mouse B cell compartment (19, 20). The rearranging VRC01 models have been widely adopted by the HIV vaccine model field (21–25).

We further adapted our complete VRC01 class mouse model to generate a new type of humanized antibody discovery mouse model that generates a primary humanized B cell receptor repertoire exclusively from a single human V_H_1-2 and, dominantly, from a human Vκ1-33, each in association with an immense diversity of CDR3s (26). The theory was that this single-V_H_1-2/Vκ1-33-rearranging model would express a large number unique CDR3-based precursors expressing human V_H_1-2/Vκ1-33 BCRs, some of which would rarely be generated in humans and frequently not be present in standard human antibody discovery mice (18). Based on that premise, and findings of prior experiments (27), it was hypothesized that immunizing this model with the SARS-CoV-2 D614G prototype spike protein might elicit unique SARS-CoV-2-neutralizing antibodies that would function through CDR3-influenced binding interactions (26). Indeed, this immunization elicited several SARS-CoV-2 neutralizing humanized antibodies of which one, SP1-77, potently neutralized all variants of concern known at the time through Omicron BA.2.754 via an interaction with SARS-CoV-2 receptor binding domain (RBD) that occurred by a dominantly HC-CDR3-based mechanism (26). While the SP1-77 neutralization was escaped by mutations in further downstream Omicron variants, the findings of that study provided proof of principle that mice with BCR repertoires derived from rearrangements of single human V_H_ and V_L_ gene segments can generate diverse antibodies against newly arising pathogens (26).

## Results

### Generation of V_H_1-2-Rearranging Mice With Diverse, Long, Human-Like HC-CDR3s.

**Fig. 1.**
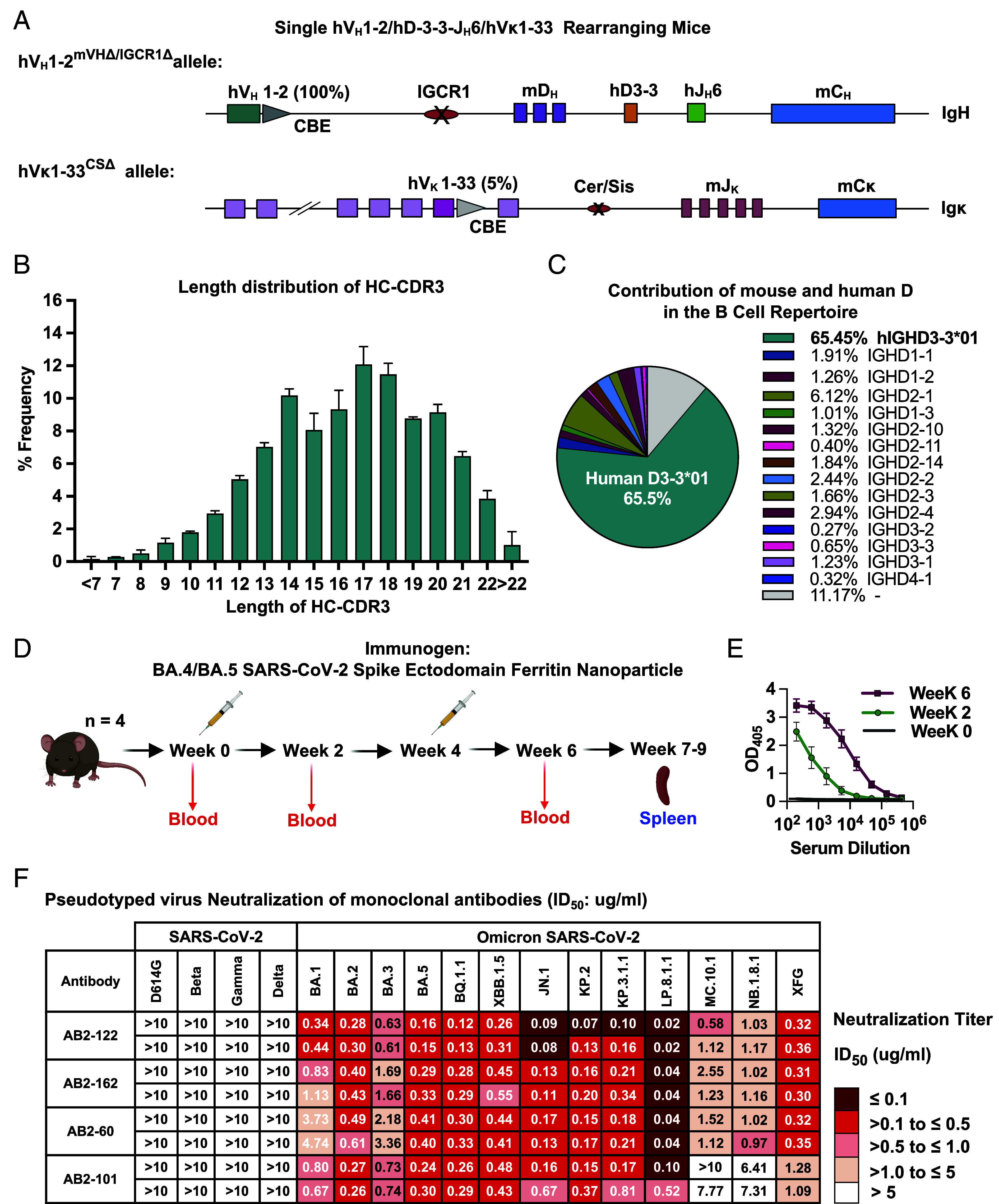
Immunization of a single human V_H_ and Vκ mouse model engineered to produce long, human-like HC-CDR3s, with SARS-CoV-2 BA.4/BA.5 spike-ferritin nanoparticles elicited bNAbs against Omicron subvariants. (*A*) Schematic illustration of modified *Igh* and *Ig*κ loci of V_H_1-2/ hD3-3-hJ_H_6/Vκ1-33–rearranging mice. The V_H_1-2/Vκ1-33-rearranging mouse model was made previously (26). We introduced a human D3-3-J_H_6 rearrangement cassette in place of mouse DQ52-J_H_1-4 in the original model to generate the V_H_1-2/ hD3-3-J_H_6 /Vκ1-33-rearranging mouse model. (*B*) HTGTS-V(D)J-seq analysis of the length of HC-CDR3 sequences of productive V_H_(D)J_H_ rearrangements of splenic B cells homozygous for the modified locus. Primers used were upstream to V_H_1-2 for V_H_1-2/ hD3-3-hJ_H_6/Vκ1-33 rearranging model. The distribution of HC-CDR3 lengths was plotted on the x-axis. Data are the means ± SD of three separate libraries prepared from a V_H_1-2/hD3-3-hJ_H_6/Vκ1-33 rearranging model (teal bars). (*C*) Fraction of repertoire contribution by mouse versus human D sequences in the V_H_1-2/hD3-3-hJ_H_6/Vκ1-33 rearranging model. Representative results from three independent biological replicates are shown. (*D*) Immunization scheme. Mice received a prime and a booster dose separated by 4 wk. (*E*) Binding responses of sera from the BA.4/BA.5 SARS-CoV-2 spike ectodomain ferritin nanoparticle-immunized V_H_1-2/hD3-3-hJ_H_6/Vκ1-33 mice at weeks 0, 2, and 6 with BA.4/BA.5 SARS-CoV-2 spike. Data represent mean ± SD from four mice. (*F*) Table shows the neutralization activities [ID_50_ (µg/mL)] of four monoclonal antibodies identified from V_H_1-2/hD3-3-hJ_H_6/Vκ1-33 against variants in pseudovirus neutralization assays performed in Huh-7 cells. ID_50_ values are color-coded according to the scale shown on the *Right*. Data of two independent experiments are shown.

To further test ability of single human V_H_ and V_L_ rearranging mice as an antibody discovery model, we engineered a modified version of our original single-V_H_1-2/Vκ1-33-rearranging mouse model (26) in which we introduced human D3-3-J_H_6 rearrangement cassette (28) in place of the proximal mouse D (DQ52) and J_H_1-4 segments in the original model to generate the “single V_H_1-2/hD3-3-hJ_H_6/Vκ1-33” rearranging mouse model (Fig. 1*A* and *SI Appendix*, Fig. S1*A*). We note that introduction of hD3-3-hJ_H_6 rearrangement cassette in this location was predicted to allow it to become an integral part of a new *Igh* V(D)J recombination center (RC) (29). The hD3-3-hJ_H_6 junctional combination also can encode much longer HC-CDR3 sequences in humans than are encoded by any mouse D and J_H_ combinations, and thus could provide a new variation of the single-V_H_1-2/Vκ1-33-rearranging mouse model that provides both a wider length range and a largely distinct set of human HC-CDR3s. Like the prior model, this new model expresses human TdT to further diversify LC-CDR3. As both mouse models have single human V_H_1-2 and *hTDT* expression, we do not repeat those terms in the names of the models hereafter, for brevity.

Flow cytometric analyses of the V_H_1-2/ hD3-3-hJ_H_6 /Vk1-33-rearranging mouse splenic B, and T cell populations revealed overall profiles broadly similar to those of wild-type mice, with quantitative analysis indicating significantly reduced B cell numbers and modestly, but significantly, increased T cell numbers (*SI Appendix*, Fig. S1*B*). Increased T cell numbers in mice with reduced B cell numbers has been previously reported (30). The contribution of human V_H_1-2 and Vκ1-33 to the splenic B cell receptor (BCR) repertoire were, respectively, 100% (as there are no other V_H_s in this mouse line) and 5% (*SI Appendix*, Fig. S1*C*). HC-CDR3 length and D usage in splenic B cells in the V_H_1-2/hD3-3-hJ_H_6/Vκ1-33–rearranging mouse model were assessed by the HTGTS-V(D)J-seq (31), employing a human V_H_1-2 bait (Fig. 1*A*). Homozygous V_H_1-2/hD3-3-hJ_H_ 6/Vκ1-33-rearranging mice generated HC-CDR3s with a peak length of 17 to 18 amino acids (Fig. 1*B*), in contrast to the original VRC01 model in which mouse Ds and J_H_s generated a peak HC-CDR3 length of 11 amino acids (26). Approximately 65% of splenic B cells from the V_H_1-2/hD3-3-hJ_H_6/Vκ1-33-rearranging mice employed the human D3-3 in association with hJ_H_6 in their productive V(D)J rearrangements (Fig. 1*C*). Predominant usage of human D3-3 in V_H_1-2(D)J_H_6 rearrangements, despite the full complement of mouse Ds upstream, may reflect its RC location, which permits human D3-3 to J_H_6 rearrangement by diffusion (32). Deletion of the IGCR1 RAG-scanning impediment on this allele, which reduces levels of upstream D rearrangements effected by loop extrusion-mediated RAG scanning (33–37), may also contribute to increased D3-3 to J_H_6 rearrangement levels. Analysis of HC-CDR3s in the primary BCR repertoires of V_H_1-2/hD3-3-hJ_H_6/Vκ1-33-rearranging mouse revealed a highly diverse V_H_1-2-based HC-CDR3 repertoire (*SI Appendix*, Fig. S1*D*). We employed the same Igκ locus configuration in the new model as that of our earlier model (26). As expected, human Vκ1-33 to mouse Jκ rearrangements generated diverse Igκ CDR3s with a peak length of 9 amino acids (*SI Appendix*, Fig. S1 *E* and *F*).

### BA.4/BA.5 Spike Immunization of V_H_1-2/hD3-3-hJ_H_6/Vκ1-33 Mice Elicited Potent Omicron bNAbs.

We immunized four V_H_1-2/hD3-3–hJ_H_6/Vκ1-33 mice twice, 4 wk apart, with BA.4/BA.5 SARS-CoV-2 spike-Ferritin nanoparticles (Fig. 1*D**; see methods for details*). All four immunized mice had strong IgG responses to BA.4/5 spike at 2 and 6 wk postimmunization (Fig. 1*E*). At 3 to 5 wk postboost, 96 BA.4/5 spike-specific IgG^+^ splenic B cells per mouse were FACS-sorted, and their IgH and Igκ variable regions were amplified by RT-PCR and sequenced (*SI Appendix*, Fig. S1*G*). These immunizations elicited multiple BA.4/BA.5 spike-specific humanized antibodies, which we categorized into four distinct lineages, as defined by distinct CDR3 sequences associated with V_H_1-2 and Vκ1-33. Each of these antibody lineages came from a different mouse (*SI Appendix*, Fig. S1*G*). We selected one antibody from each lineage (termed AB2-122, AB2-162, AB2-60, and AB2-101) based on having the highest level of SHM at the amino acid level (*SI Appendix*, Fig. S1*H*). For each, we expressed the *Igh* V(D)J exon with human IgG1 or IgG4 and the *Ig*κ VJ exon with a human Igκ constant region. Pseudovirus neutralization assays demonstrated that none of these antibodies neutralized the prototype D614G virus or earlier pre-Omicron variants, including Beta, Gamma, and Delta (Fig. 1*F* and *SI Appendix*, Fig. S1*I*). However, all four of these antibodies neutralized most tested Omicron subvariants beyond BA.1 and potently neutralized recent Omicron variants from JN.1 through LP8.1.1, with AB2-122 being the most highly potent (Fig. 1*F* and *SI Appendix*, Fig. S1*I*). The MC.10.1 variant, however, was not neutralized by AB2-101 and only weakly neutralized by AB2-122, AB2-162, and AB2-60 (Fig. 1*F**).* Similarly, the NB.1.8.1 variant was not neutralized by AB2-101 and weakly neutralized by AB2-122, AB2-162, and AB2-60 (Fig. 1*F*). Finally, the recent XFG variant was weakly neutralized by AB2-101 and relatively potently neutralized by AB2-122, AB2-162, and AB2-60 (Fig. 1*F*).

### Related HC-CDR3s Underlie Common RBD Epitope Targeting by Omicron bnAbs.

**Fig. 2.**
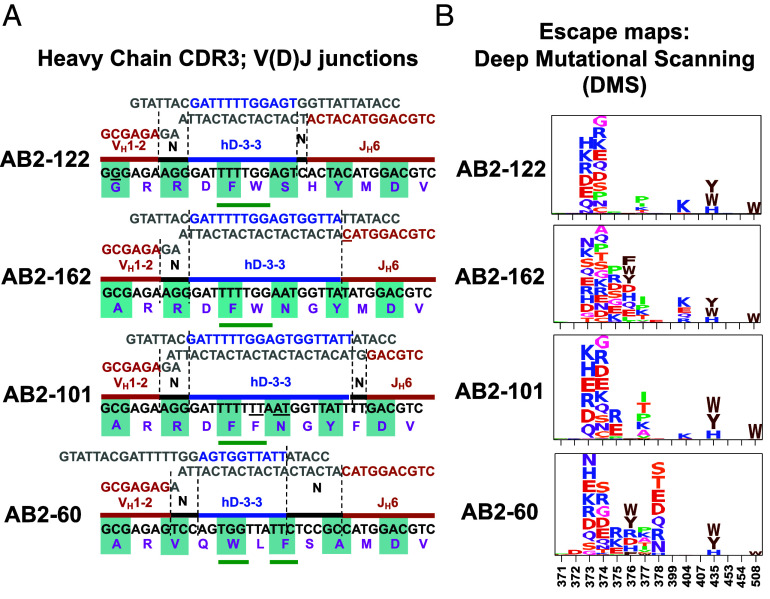
Single- V_H_1-2/hD3-3-hJ_H_6/Vκ1-33 mice elicit Omicron bNAbs with conserved two aromatic amino acids in the HC-CDR3 and target the same RBD epitope. (*A*) Nucleotide and amino acid sequences of HC-CDR3 V(D)J junctions from Omicron bNAbs (AB2-122, AB2-162, AB2-101, and AB2-60) isolated from V_H_1-2/hD3-3-hJ_H_6/Vκ1-33 mice. In the *Top* portion of each diagram, the V_H_ (red), N, hD3-3 (blue), and hJ_H_6 sequences (orange) that contribute to each of the four junctions are indicated, and their contribution to the overall HC-CDR3 sequence is shown in the color-coded line in the center. The nucleotide sequence of each HC-CDR3 is shown in black just below the color-coded HC-CDR3 diagram, and the encoded amino acid sequence is shown in purple at the *Bottom* using standard AA terminology. Two adjacent aromatic residues centrally located within the HC-CDR3 are highlighted (green underline) for three HC-CDR3 sequences, and in another aromatic residues separated by leucine are shown (see text for details) (*B*) Deep mutational scanning (DMS) escape maps for the corresponding antibodies, shown as logo plots across the RBD. Amino acids are colored according to chemical properties, and letter height in the logo plots indicates escape scores. More details on DMS maps have been described (38).

Comparative sequence analyses of the CDR3s of AB2-122, AB2-162, AB2-101, and AB2-60 antibodies (Fig. 2*A*) revealed that, despite the V_H_1-2/hD3-3–hJ_H_6/Vκ1-33 model generating predominantly longer, human-like HC-CDR3s, the four independent Omicron-neutralizing antibodies each had 12AA HC-CDR3s (Fig. 2*A*), which are much shorter than the 17AA average length of HC-CDR3s generated by this model (Fig. 1*B*). Although all four antibodies utilize the human D3-3 and J_H_6 gene segments in their HC-CDR3s, they incorporate different portions of these segments, resulting in distinct HC-CDR3s. Yet, these four antibodies have related HC-CDR3 features. In this regard, three of four had two adjacent aromatic amino acids in the same HC-CDR3 positions, even though these two residues were encoded by different regions of human D3-3 and, in 2 cases (AB2-162 and AB2-101), generated by somatic hypermutation; in AB2-122, these residues are encoded by the D3-3 segment (Fig. 2*A*). The other antibody has an aromatic tryptophan and a leucine in the same positions, followed by an aromatic acid. Leucine is an aliphatic hydrophobic residue that is sufficiently bulky to substitute for phenylalanine to some extent, indicating a preference for bulky hydrophobic residues at the center of HC-CDR3. Therefore, all 4 HC-CDRs, are 12AA in length with a pair of aromatic AAs in the same HC-CDR3 position, or in one case a pair of aromatic/aliphatic hydrophobic AAs, in this same HC-CDR3 position (Fig. 2*A*). The LC-CDR3s of all four antibodies were 9 amino acids long, which is the most common length encoded by the V_H_1-2/hD3-3-hJ_H_6/Vκ1-33 model with no notable other common features (*SI Appendix*, Fig. S1 *F* and *I*).

To test the RBD epitope(s) bound by these four antibodies, we used deep mutational scanning (DMS) assays (38) to systematically map the impact of RBD mutations on antibody binding. This analysis revealed that the four antibodies shared highly similar escape profiles, with critical escape mutations clustering within the RBD 373 to 378 region (Fig. 2*B*), suggesting all four belong to class 4 or F1 epitope group and share similar RBD-binding modes. All four bNAbs are primarily evaded by mutations at residues 373, 374, and 435 of the KP.3 RBD (Fig. 2*B*). AB2-162, AB2-101, and AB2-60 were also affected by additional mutations at 375, 376, and 377 residues. At the same time, AB2-122 was less affected by these mutations (Fig. 2*B*). AB2-60, in addition to the mutations mentioned above, is uniquely sensitive to substitutions at position 378. This sensitivity may be due to its HC-CDR3 sequence containing a leucine residue in place of an aromatic hydrophobic residue, thereby preserving hydrophobic bulk but eliminating aromatic π–π interactions that could stabilize epitope binding (Fig. 2 *A* and *B*). Overall, DMS data suggest that all four antibodies recognize a common epitope. Notably, AB2-122 stands out as the most escape-resistant antibody, maintaining binding despite mutations that compromise the other three.

### AB2-122 bNAb Targets the RBD Epitope Via Two Adjacent Aromatic Residues in its HC-CDR3.

**Fig. 3.**
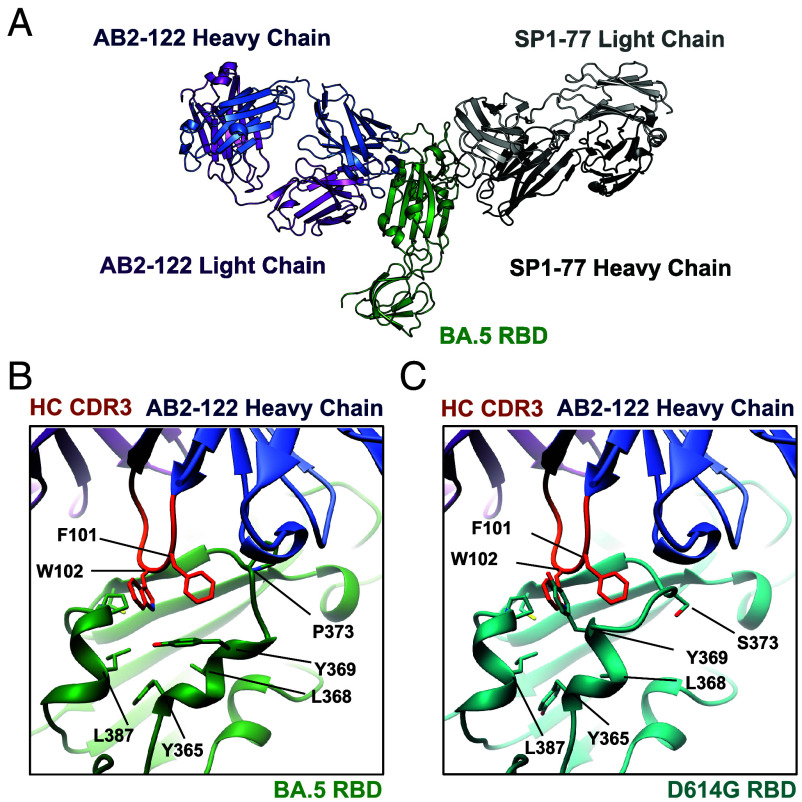
AB2-122 targets the RBD epitope via two adjacent aromatic amino acid residues in its HC-CDR3. (*A*) Cryo-EM structure of the Omicron BA.5 S1 monomer bound to AB2-122 and SP1-77 Fabs shown in ribbon diagram, with the BA.5 RBD in green, AB2-122 heavy and light chains (LCs) in blue and magenta, and SP1-77 heavy and light chains are in dark and light gray, respectively. (*B*) Close-up view of the interaction between AB2-122 Fab and the BA.5 RBD. The HC-CDR3 of AB2-122 is highlighted in orange, with Phe101 and Trp102 in stick model latched onto the hydrophobic patch on the BA.5 RBD. The contacting residues of the BA.5 RBD, including Y365, L368, Y369, P373, and L387, are also shown in stick model. The S373P mutation in BA.5 reconfigures the helix^364–372^, exposing a hydrophobic patch that enables Phe101-Trp102 of AB2-122 in the HC-CDR3 to bind. (*C*) Close-up view of the modeled interaction between AB2-122 Fab and the D614G RBD (in cyan). In D614G, the unshifted helix^364–372^ of the RBD clashes with the HC-CDR3 of the antibody.

To further elucidate the epitope of the antibodies on the RBD and the relative contributions of V_H_ or V_L_ CDR1 and CDR2 versus CDR3s in epitope recognition, we determined the structure of AB2-122 in complex with Omicron BA.5 spike by cryoelectron microscopy (cryo-EM) (Fig. 3*A*). The spike consists of noncovalently linked receptor-binding subunit S1 and fusion subunit S2. When AB2-122 was mixed with the full-length spike trimer, it induced S1 dissociation from the trimer, similar to what was observed with soluble ACE2 (39). Incubation of the spike trimer with SP1-77 and AB2-122 Fab fragments led to a complex of monomeric S1 with both antibodies that would facilitate particle alignment for cryo-EM analysis (*SI Appendix*, Fig. S2*A*). Consequently, the AB2-122-S1-SP1-77 stable complex was used for structure determination, and its structure was refined to 3.0 Å resolution (*SI Appendix*, Fig. S2 *B* and *C*). The cryo-EM structure of Omicron BA.5 spike complexed with AB2-122 has revealed that it binds to an epitope of the RBD on the opposite side of the SP1-77 epitope, near the class 4 or F1 epitope and only accessible when the RBD is in the up conformation (40) (Fig. 3*A*). Notably, the binding interface of AB2-122 is dominated by its HC-CDR3, consistent with the proposed selection for CDR3-centric antibodies in the V_H_1-2/hD3-3-hJ_H_6/Vκ1-33 rearranging model (Fig. 3*B*). Two aromatic residues, Phe101 and Trp102, in the AB2-122 HC-CDR3, make key contacts with the RBD by latching onto an Omicron-specific RBD hydrophobic patch formed by residues 365 to 387(Fig. 3*B*), and interacting extensively with Y365, L368, Y369, P373, and L387 of the RBD (Fig. 3*B*). We note that a founding S373P mutation in the Omicron spike *(**SI Appendix*, Fig. S3*A*) appears to rigidify the local polypeptide chain and cause a small shift of the helix^364–372^, which has created the epitopes targeted by this set of antibodies (Fig. 3*B*). Molecular modeling suggested that the helix^364–372^ in early SARS-CoV-2 variants, including D614G, would sterically clash with the two aromatic residues at the tip of the HC-CDR3 loop of these antibodies (Fig. 3*C*), explaining lack of neutralization against pre-Omicron variants (Fig. 1*F*).

**Fig. 4.**
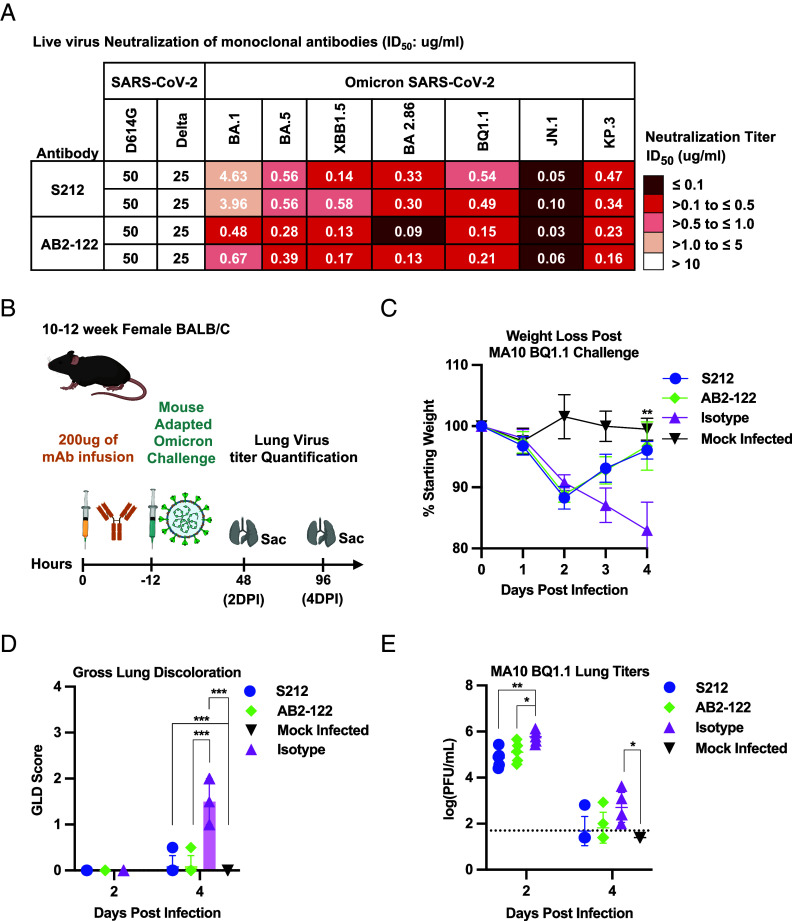
Prophylactic treatment of mice with Omicron bNAbs from rearranging mouse models protects against challenge with an Omicron subvariant. (*A*) Neutralization activity of AB2-122 and S212 against SARS-CoV-2 variants of interest, (VOIs), was determined by live-virus neutralization assays. Inhibitory concentration at 50%, IC_50_, Values are color-coded according to the scale shown at right. Data of two independent experiments are shown. (*B*) Experimental design for prophylactic antibody administration and viral challenge. Mice were administered 200 μg of either AB2-122, S212, or CH65 control antibody intraperitoneally (i.p.). Twelve hours after antibody administration, all treated animals were challenged intranasally with 10^5^ PFUs of MA10 BQ.1.1 in 50 μL PBS. An additional group (n = 5) received PBS alone and served as a mock infection control. (*C*) Percent weight change following challenge with MA10 BQ.1.1 in mice prophylactically treated with Omicron bNAbs (AB2-122 or S212) or the CH65 control antibody. Weight change was calculated relative to baseline (day 0) for each animal. Data are means ± SD. Statistical analysis used two-way ANOVA followed by Tukey’s multiple comparisons test, comparing each experimental group to the CH65 control. (*D*) Gross lung discoloration scores on days 2 and 4 postinfection. Lung discoloration was scored after euthanizing the animals on a scale of 0-4, with 4 being the most severe according to the University of North Carolina Chapel Hill Institutional Biosafety Committee and Institutional Animal Care and Use Committee under protocol 23–085. Data are presented as dot plots with bars indicating the mean. Statistical analysis used one-way ANOVA and Tukey’s multiple comparisons test. (*E*) Lung viral titers following MA10 BQ.1.1 infection. Infectious virus in lung tissue was quantified by plaque assay at days 2 and 4 postinfection. Data are means ± SD. Statistical analysis used one-way ANOVA and Tukey’s multiple comparisons test. *P < 0.05; **P < 0.01.

To assess whether AB2-122 interacts similarly with more recent Omicron subvariants, we also determined the structure of AB2-122 in complex with the KP.3.1.1 Spike, using BD57-2704 (41) as a noncompeting helper antibody to facilitate alignment in cryo-EM (*SI Appendix*, Fig. S4*A*). The KP.3.1.1 RBD model shown corresponds to a locally refined reconstruction. The interactions of AB2-122 with KP.3.1.1 RBD mirror those between the antibody and BA.5 RBD (Fig. 4 *A* and *B*), with the binding dominated by the two HC-CDR3 aromatic residues, as expected (*SI Appendix*, Fig. S4*B*). There are no significant differences in the RBD conformation or Fab orientation between the AB2-122-KP.3.1.1 and AB2-122-BA.5 complexes (*SI Appendix*, Fig. S4 *B* and *C*), ruling out any obvious structural changes caused by the helper antibodies, and confirming a consistent mechanism of epitope targeting by AB2-122 across multiple Omicron subvariants. Not surprisingly, alanine substitution of Phe101 and Trp102 in AB2-122 resulted in substantial loss of its binding activity, further supporting the role of these aromatic residues in the HC-CDR3 in recognizing the Omicron RBD *(**SI Appendix*, Fig. S3*B*).

### AB2-122 Neutralizes SARS-CoV-2 Omicron Variants by Blocking ACE2 Receptor Binding.

DMS and structural data indicate that the neutralization by AB2-122 antibody involves inhibiting virus binding to the ACE2 receptor. In this context, surface plasmon resonance competition assays demonstrated that AB2-122 antibody blocked the binding of the RBD to the ACE2 receptor (*SI Appendix*, Fig. S5 *A* and *B*). SPR competition and neutralization assays with AB2-122 IgG and its corresponding Fab *(**SI Appendix*, Fig. S5 *C* and *D*) revealed that Fab of AB2-122 blocked ACE2 binding in the same way as the full IgG antibody (*SI Appendix*, Fig. S5 *B*–*D*). Moreover, the superposition of the AB2-122-BA.5 S1 complex with the ACE2–RBD complex by the RBD shows that the AB2-122 Fab sterically clashes with ACE2, primarily through its HC, further suggesting blocking viral RBD interaction with ACE2 as a mechanism of neutralization (*SI Appendix*, Fig. S5*E*).

### V_H_1-2/Vκ1-33 Mouse Model Generates Highly Related Omicron-Neutralizing Antibodies.

For comparison, we also immunized the hV_H_1-2/HVκ1-33-rearranging mouse model (26) (*SI Appendix*, Fig. S6*A*), which uses mouse Ds and J_H_s with BA.4/BA.5 SARS-CoV-2 spike-ferritin nanoparticles (*SI Appendix*, Fig. S6*B*). The hV_H_1-2/HVκ1-33-rearranging mouse model generates diverse mouse-like HC-CDR3s with a median length of 11AAs (26). All mice showed strong IgG responses at 2- and 6-weeks postimmunization (*SI Appendix*, Fig. S6*C*), allowing us to identify four distinct antibodies (S212, S77, L44, and L52) that potently neutralized most tested Omicron variants (except BA.1), but not pre-Omicron strains (*SI Appendix*, Fig. S6 *D–F*). While the neutralization pattern of these Abs was reminiscent of those isolated from the V_H_1-2/hD3-3-hJ_H_6/Vκ1-33 model, they had somewhat reduced potency against more recent subvariants that were tested with both sets of antibodies (*SI Appendix*, Fig. S6*F*). Notably, each of these antibodies had 12AA HC-CDR3s, with a pair of adjacent aromatic AAs in its center *(**SI Appendix*, Fig. S7*A**)* and exhibited escape profiles in DMS assays that were highly related to those from the V_H_1-2/hD3-3-hJ_H_6/Vκ1-33 model (*SI Appendix*, Fig. S7*B*), indicating that they target the same general Omicron epitope. Furthermore, cryo-EM structures of the S1 complex with S212 or L52 antibody (*SI Appendix*, Figs. S8 *A*–*D*, S9*A*, S10 *A*–*D*, and S11*A*), which had the most divergent HC-CDR3s, confirmed that their binding to RBD is very similar to those from V_H_1-2/hD3-3-hJ_H_6/Vκ1-33 rearranging model (*SI Appendix*, Figs. S9 *B* and *C* and S11 *B* and *C*). Like V_H_1-2/hD3-3-hJ_H_6/Vκ1-33 derived antibodies, these antibodies are dependent on two centrally located two aromatic residues to bind the RBD (*SI Appendix*, Fig. S12 *A*–*C*). Similar to V_H_1-2/hD3-3-hJ_H_6/Vκ1-33 model-derived antibodies, these antibodies likely function by blocking ACE2 receptor Binding (*SI Appendix*, Figs. S13 *A*–*D* and S14 *A* and *B*). Overall, our combined findings suggest that Omicron-specific mutations have generated an Omicron-specific binding epitope that dominantly elicited all eight independently isolated Omicron-variant neutralizing antibodies from two independent mouse models.

### AB2-122 and S212 Protect Mice from Omicron BQ1.1 Challenge.

Before assessing protective efficacy, we tested the two most potent Omicron bnAbs-AB2-122 from the V_H_1-2/hD3-3-hJ_H_6/Vκ1-33 model and S212 from the V_H_1-2/Vκ1-33 model for their ability to neutralize replication-competent live viruses. Similar to pseudovirus neutralization, AB2-122 and S212 antibodies neutralized all Omicron variants tested, and did not neutralize the prototype D614G virus or pre-Omicron variant Delta (Fig. 4*A*). S212 weakly neutralized the BA.1 variant, consistent with its activity in the pseudovirus assay. In contrast, AB2-122 more robustly neutralized BA.1 in the replication-competent assay as compared to S212 (Fig. 4*A*). To test the protective efficacy of our bnAbs, we evaluated AB2-122 and S212 in vivo using a lethal Omicron challenge model. We prophylactically administered AB2-122 or S212 twelve hours before viral challenge. Mice received 200 μg of AB2-122 (n = 10), S212 (n = 10), or an isotype control antibody (CH65; n = 10) via intraperitoneal injection (i.p.). All animals were subsequently intranasally challenged with 10^5^ PFUs of mouseadapted Omicron BQ1.1 (MA10 BQ1.1) (42). PBS mock-infected mice (n = 5) served as a sham control. Mice were euthanized on day 2 or 4 postinfection and lung tissues were harvested for gross pathology assessment and viral load quantification by plaque assay (Fig. 4*B*) according to the University of North Carolina Chapel Hill Institutional Biosafety Committee and Institutional Animal Care and Use Committee under protocol 23–085. Treatment with AB2-122 or S212 antibodies resulted in significantly reduced weight loss relative to CH65 treated controls at day 4 postinfection (Fig. 4*C*). While CH65-treated animals showed significantly higher gross lung discoloration (GLD) scores, consistent with substantial lung pathology, AB2-122 or S212 bnAbs treated mice had lung GLD scores comparable to those of mock-infected controls (Fig. 4*D*), indicating protection against viral induced lung pathology. Viral titers quantified in lung tissues on days 2 and 4 postinfection revealed reduced viral loads in AB2-122 and S212-treated mice as compared with CH65-treated controls (Fig. 4*E*). Together, these results demonstrate that both bnAbs confer comparable protection against SARS-CoV-2 Omicron BQ1.1 lethal challenge in vivo.

## Discussion

We have described a new V_H_1-2/hD3-3-hJ_H_6/Vκ1-33–rearranging mouse model and demonstrated its utility for the discovery of antibodies that neutralize recent SARS-CoV-2 Omicron variants. In the new model, human D3-3-to-J_H_6 rearrangements contributed to 65% of the DJ_H_ junctions of the V(D)J repertoire, as compared to the fully mouse-encoded DJ_H_ junctions in the prior V_H_1-2/Vκ1-33-rearranging model. Thus, in the V_H_1-2/hD3-3-hJ_H_6/Vκ1-33–rearranging model, a large fraction of the peripheral B cells express diverse HC-CDR3s fully generated by V(D)J recombination of the introduced human V_H_, D, and J_H_ sequences. Moreover, the V_H_1-2/hD3-3-hJ_H_6 /Vκ1-33-rearranging model generated a HC-CDR3 repertoire that had a substantially increased average HC-CDR3s length compared to the V_H_1-2/Vκ1-33-rearranging model. We immunized both the V_H_1-2/hD3-3-hJ_H_6/Vκ1-33–rearranging and previously described V_H_1-2/Vκ1-33-rearranging mouse models with the same Omicron BA.4/5 spike-ferritin nanoparticles. Upon immunization, each model yielded four independent antibodies that broadly and potently neutralized most downstream Omicron variants. Surprisingly, the Omicron bnAbs from both models had 12AA HC-CDR3s, despite the new model predominantly generating much longer CDR3s. In addition, all but one of bNAbs from both models contained pairs of adjacent aromatic residues in the center of their HC-CDR3s, with four out of 7 generated by SHM and others encoded by different regions of the long human D3-3 employed by the new model. All 8 of these Omicron bnAbs targeted the same conserved Omicron-specific epitope as defined by both DMS and cryo-EM analysis. Moreover, representative antibodies from both models function by blocking RBD binding to ACE2 receptor and protect mice from Omicron BQ1.1 challenge. Thus, all 8 Omicron bnAbs isolated from the hV_H_1-2/hVκ1-33 model and the V_H_1-2/ hD3-3-hJ_H_6 /Vκ1-33–rearranging model converged on the same structural solution, independently selecting functionally similar HC-CDR3s optimized to target the same, or very similar, conserved Omicron-specific epitope.

Class 4 SARS-CoV-2-neutralizing antibodies are a structurally defined group of RBD-directed antibodies that recognize a conserved, cryptic epitope on the inner face of the SARS-CoV-2 RBD, distal from the ACE2-binding site (40). DMS studies have grouped antibodies that engage this same region into what is referred to as the F1 group (38). The F1 group was further divided into F1.1 and F1.2 subgroups (41). F1.1 subgroup SARS-CoV-2 neutralizing antibodies target a traditional Class-4 cryptic inner-face site; whereas, F1.2 binds an adjacent but RBM-proximal epitope on the surface of the RBD (41). F1.1 antibodies are largely nonneutralizing with respect to Omicron variants and commonly induced by WT spike vaccination, whereas F1.2 antibodies, which are all elicited by Omicron infection, show a broad range of neutralization of Omicron-lineage variants (41). Notably, the F1.2 Omicron-specific human antibodies, based on DMS patterns, bind the same or a very similar epitope to that of the 8 Omicron bnAbs we describe in this study (41). While the F1.2 human Omicron-neutralizing antibodies bind a similar epitope as the eight isolated from our V_H_1-2-rearranging mouse models, the F1.2 human antibodies employ a diverse set of V_H_s and V_L_s, raising the possibility that these human antibodies may also feature HC-CDR3 dominance in promoting specific epitope binding. The recent Omicron MC.10.1 variant largely escaped the four Omicron bnAbs derived from the V_H_1-2/hD3-3-hJ_H_6/Vκ1-33 mouse model although three retained weak neutralization (Fig. 1*F*), As all four of these antibodies neutralized KP.3.1.1, which only differs by an A435S substitution from MC.10.1, it is likely that this mutation, which lies in the binding epitope (Fig. 2*B*), is responsible for the observed escape. Notably, however, the A435S mutation of the MC.10.1 variant also reduced its fitness, suggesting that this mutation may represent a trade-off for MC.10.1 proliferation in escaping antibodies that target this epitope (43). Similarly, the Omicron NB.1.8.1 variant also carries the A435S mutation (44), which may explain its similar neutralization activity as compared to MC.10.1 (Fig. 1*F*). On the other hand, the Omicron XFG variant originated from a different set of mutations (44), none of which lie in the F1.2 binding epitope, therefore allowing AB2-122, AB2-162, and AB2-60 maintain relatively potent neutralization of XFG (Fig. 1*F*).

Early Omicron mutations created a new immunogenic site that has highly selected similar antibodies from both hV_H_1-2/hVκ1-33 and the V_H_1-2/hD3-3-hJ_H_6/Vκ1-33 mouse models, despite the latter producing antibodies with substantially longer HC-CDR3s. Under immune pressure, SARS-CoV-2 continues to evolve, giving rise to many different variants. Except for a small shift of helix^364–372^ in Omicron subvariants, the overall structure of the RBD has changed little among all these variants (45, 46), probably reflecting the need for the virus to keep the RBD structure intact to maintain ability to engage the ACE2 receptor without compromising viral fitness. Our study shows that the local structural rearrangement near the F1.2 epitope caused by the helix shift in Omicron created a surface-exposed hydrophobic patch. This patch is targeted by both AB2-122, S212, and L52 primarily through two aromatic residues in their HC-CDR3 based on structures, and similarly targeted by our other 5 Omicron bnAbs based on DMS and the conserved aromatic amino acid pairs in their HC-CDR3s. None of these antibodies recognized RBDs in pre-Omicron variants with an unshifted helix^364–372^ and, correspondingly, did not neutralize tested pre-Omicron variants. The 8 Omicron bnAbs we describe potently neutralize nearly all tested Omicron subvariants; albeit those from the V_H_1-2/hD3-3-hJ_H_6/Vκ1-33 model are, on average, more potent overall with several showing great potency with respect to the recent LP.8.1.1 variant. The reason for the potentially more potent neutralization by antibodies derived from the V_H_1-2/hD3-3-hJ_H_6/Vκ1-33 mouse model is unclear. However, as the potency ranges between Omicron bnAbs from the two models and RBD binding affinities marginally overlap, apparent differences could possibly reflect stochastic variation given the limited number of bnAbs isolated thus far from each model. This possibility could be addressed by immunizing greater numbers of the current mouse models, but a more efficient assessment could be provided by modifications of these two models to enforce rearrangement of single human Vκ and Jκ segments in the absence of mouse Vκ rearrangements, which theoretically would allow 20-fold more humanized bnAbs to be isolated from a single immunization. Finally, even though it is likely that Omicron variants may arise that escape all of the antibodies we describe, immunization of the mouse models described here with RBDs from putative new variants may elicit new sets of antibodies that could neutralize them and potentially others well beyond them.

## Materials and Methods

### V_H_1-2/hD3-3-hJ_H_6/Vκ1-33 Rearranging Mouse.

This model was generated by methods described previously (18, 26, 28) and modified for the new model as summarized here: All genetic modifications were introduced into ES cells (129/Sv background) derived from the inner cell mass of blastocysts (47) derived from single-V_H_1-2/Vκ1-33-rearranging mouse model (26). We used two gRNAs that target the mouse DQ52-J_H_1-4 locus (5’gctactggtacttcgatgtc3’ and 5’gattactatgctatggactac3’) to integrate, via homologous recombination, a hD3-3 and hJ_H_6 cassette (28) in place of mouse DQ52 to J_H_ 4. We confirmed the correct integration of the cassette by Southern blotting. All ES cells were cultured and maintained as previously described (26). ES clones with the correctly integrated cassette were injected into Rag2-deficient blastocysts to generate chimeras harboring the V_H_1-2/ hD3-3-hJ_H_6/Vκ1-33-cassette allele. The chimeric mice were bred with germline V_H_1-2/Vκ1-33 rearranging mice to introduce the hD3-3 and hJ_H_6 cassette allele into the germline and with further breeding to generate mice homozygous for the V_H_1-2/ hD3-3-hJ_H_6 and Vκ1-33 loci that were used for described experiments. Mice were housed as described previously (26). Splenic B and T cells from 7-week-old homozygous V_H_1-2/hD3-3-hJ_H_6/Vκ1-33 rearranging mice were characterized by staining with PE/Cy7 anti-B220 (BD Biosciences:552772); PE anti-Thy1.2 (eBioscience:2-0902-83); BV605 anti-IgM (BioLegend:406523); APC anti-IgD (BioLegend: 405714). All mouse work was approved by the Boston Children’s Hospital-IACUC (protocol 00002005).

### Purification of Splenic B cells and HTGTS-Repertoire-Sequencing Analysis.

HTGTS-V(D)J-seq libraries were generated from splenic B cell DNA(10ug) from the spleen of mice aged 4 to 7 wk as previously described (31, 48). For HC variable region exons, a bait primer that targets the human V_H_1-2 leader intron was used to capture the full-length V(D)J sequences for analysis of the variable region repertoire (49). For Igκ light-chain analyses, a mixture of bait primers targeting mouse Jκ1, Jκ2, Jκ4, and Jκ5 was used to capture the repertoire as previously described (18, 48). HTGTS Libraries were sequenced on Illumina MiSeq (2x300 bp) or NextSeq (2x150 bp) platforms and analyzed using the HTGTS-V(D)J-seq pipeline (31).

### Immunogen and Immunizations.

BA.4/.5 spike ferritin nanoparticles were prepared by conjugating the BA.4/.5 spike ectodomain to *Helicobacter pylori* ferritin nanoparticles, as previously described (50, 51) and in *SI Appendix*, *Materials and Methods*. Seven-week-old mice were immunized intraperitoneally with 25 µg antigen and 60 µg Polyinosinic-polycytidylic acid (poly(I:C)) adjuvant, following a previously described scheme (26).

### Enzyme-Linked Immunosorbent Assay (ELISA).

ELISAs were performed as previously described (52) with BA.4/5 spike protein as coating antigen (100 ng per well) and serial dilutions of sera (starting at 1:200).

### BA.4/.5 Spike-Specific B Cell Sorting and Single-Cell Reverse Transcription PCRs.

Antigen-specific IgG^+^ B220^+^ IgD^−^ B cells from immunized mouse spleens were single-cell sorted, and RT-PCR was performed as described previously (18, 26). Specifically, BA.4/BA.5 SARS-CoV-2 spike protein was fluorescently labeled with Alexa Fluor 647 using a protein labeling kit (Thermo Fisher Scientific, A30009). Other antibodies used for cell-surface staining were described previously (26). Single antigen-specific IgG+ B cells were sorted into 96-well plates, and cDNA synthesized using constant-region primers targeting Cμ, Cγ1, Cγ2a, and Cκ. Rearranged V_H_1-2 heavy-chain and Vκ1-33 light-chain exons were amplified by nested PCR and sequenced by Sanger sequencing.

### Monoclonal Antibody Production.

Antibodies were generated as described previously (26). Briefly, Antibody expression constructs encoding HC- and LC variable region exons fused to human IgG1 and Cκ constant regions, respectively, were generated by GenScript. Antibodies were expressed in Expi293F cells (Thermo Fisher) and purified from filtered supernatants by Protein A chromatography (Cytiva). Purified IgGs were buffer exchanged into PBS and used for assays.

### Spike-Pseudotyped VSV Construction and Neutralization Assays.

Spike-pseudotyped vesicular stomatitis virus (VSV) expressing the SARS-CoV-2 variant spike glycoproteins (D614G, Beta, Gamma, Delta, BA.1, BA.2, BA.3, BA.5, BQ.1.1, XBB.1.5, JN.1, KP.2, KP.3.1.1, LP.8.1.1, MC.10.1, NB.1.8.1, and XFG) was generated as previously described (53, 54). Neutralizing activity of serially diluted mAbs was assessed by preincubating the mAbs with pseudovirus before infection of Huh-7 cells, as described previously (55). Pseudovirus neutralization assays in 293 T/ACE2 cells were performed as described previously (56). Additional details can be found in *SI Appendix*, *Materials and Methods*.

### DMS.

To map antibody escape, we constructed yeast-displayed RBD mutant libraries (XBB.1.5 and KP.3) as before (57, 58). Libraries were incubated with mAbs and sorted by FACS to enrich mutants with reduced binding. Escape mutations were identified by barcode sequencing, and escape scores were calculated as described previously (41). Additional details can be found in *SI Appendix*, *Materials and Methods*.

### Cryo-EM analysis.

The full-length Omicron BA.5 spike protein was produced via a protocol described previously (59), and used to prepare the antibody–spike complexes for cryo-EM studies. Selected cryo-EM grids were used to acquire images with a Titan Krios transmission electron microscope (Thermo Fisher Scientific). Particle picking, two-dimensional (2D) classification, three-dimensional (3D) classification, and refinement were carried out in cryoSPARC v.3.3.2 (60). Model building was performed in Coot (61), and iterative refinement in Phenix (62) and ISOLDE (63). Model validation was conducted using Phenix and Molprobity (64). Additional details, including AB2-122-KP.3.1.1 spike complex cryo-EM analysis, are provided in the *SI Appendix*, *Materials and Methods*.

### Fab Preparation and SPR Competition Assay.

Fab fragments were generated as described previously (65). Briefly, AB2-122, S212, and S77 IgGs were digested with papain-agarose resin (Thermo Fisher Scientific) and the Fab fragments were then separated with rProtein A Sepharose Fast Flow resin (Cytiva). Residual cysteine was removed by centrifugal filtration (65). Fab quality was determined by SDS-PAGE under reducing and nonreducing conditions. Size-exclusion chromatography done by using a Superdex 200 Increase 10/300 column(Cytiva). SPR Competition assays were performed as described before (66). Briefly, Sequential injections of Omicron XBB.1.5 _ RBD protein were used at 20 nM over immobilized mAb or Fab and were immediately followed by analyte mAb, Fab, or ACE-2 (200 nM). The dissociation phase was followed by regeneration of the immobilized surface. Double reference subtraction was used to account for signal drift. Data analyses were performed with Biacore S200 evaluation software (Cytiva) (66).

### Live-Virus Neutralization Assays and In Vivo Omicron Challenge Studies.

Live-virus assays were performed as previously described (67, 68). For in vivo studies, Female BALB/cAnNHsd mice (Envigo strain 047) were obtained at 8-10 wk old and housed under standard conditions. 12 h prior to infection, mice received 200 μg of antibody via intraperitoneal injection. Mice were infected intranasally with 10^5^ PFU SARS-CoV-2 MA10 BQ1.1 (MA10 BQ1.1) in 50 μl PBS under ketamine–xylazine anesthesia, as previously described (42). Mice were weighed daily over the course of infection. On days 2 and 4 postinfection mice from each treatment group (n = 5/timepoint) were euthanized for tissue collection and gross lung discoloration (GLD) scoring, which indicates lung hemorrhage and/or congestion on a semiquantitative scale ranging from 0 (least severe) to 4 (most severe) based on the intensity and extent of discoloration. The right inferior lung lobe was collected for measurement of viral titer from individual animals, as previously described (42). Additional details are in *SI Appendix*, *Materials and Methods*. All animal work was approved by University of North Carolina Chapel Hill Institutional Biosafety Committee and Institutional Animal Care and Use Committee under protocol 23 to 085. All infections and downstream assays were performed at ABSL3 in accordance with Environmental Health and Safety. All work was performed with approved standard operating procedures and safety conditions for SARS-CoV-2, conforming to the safety requirements recommended by BMB, HHS, CDC, and the NIH.

### Statistical Analysis.

Data were analyzed using statistical tests mentioned in the figure legends in GraphPad Prism (v. 10.6.1).

## Supplementary Material

Appendix 01 (PDF)

## Data Availability

High-throughput sequencing data have been deposited in ArrayExpress (E-MTAB-16106) (69). All study data are included in the article and/or *SI Appendix*.
