# Supplementary material for: Recurrent SARS-CoV-2 Omicron broadly neutralizing humanized antibodies in different single human VH1-2-rearranging mouse models
Source: Proc Natl Acad Sci U S A. 2026 Mar 23;123(13):e2537053123. doi: 10.1073/pnas.2537053123 (PMC13037937; doi:10.1073/pnas.2537053123)
Supplement: Supplementary file 1 — Appendix 01 (PDF) [file pnas.2537053123.sapp.pdf]

**Supporting Information for**

# Recurrent SARS-CoV-2 Omicron Broadly Neutralizing Humanized Antibodies in Different Single Human $V_H$1-2-Rearranging Mouse Models

Himanshu Batra[a,b,c,,1,2], Sai Luo[a,b,c,1,2,3], Kevin O. Saunders[d,e,f,2], Jaclyn S. Higgins[g,2], Fanchong Jian[h,i], Jun Zhang[j,k,4], Md Golam Kibria [j,k,5], GM Jonaid[j,k], Qingchen J Zhou[a,b,c], Amanda Eaton[d,e], Kenneth Cronin[d,l], Michael L Mallory[g], Melissa Mattocks[g], Robert J. Edwards[d], Robert Parks[d,e], Esther M. Lee[d,f], Adam Yongxin Ye[a,b,c], Aimee Chapdelaine Williams[a,b,c], Geeyoun Jung[a,b,c], Katayoun Mansouri[d], S.Munir Alam[d,e], David C. Montefiori[d,e], Ming Tian[a,b,c], Ralph S. Baric[g,6], Yunlong Cao[h,i,6], Barton F. Haynes[d,e,m,6], Bing Chen[j,k,6], Frederick W. Alt[a,b,c1,6]

[a]Howard Hughes Medical Institute, Boston Children's Hospital, Boston, MA 02115; [b]Program in Cellular and Molecular Medicine, Boston Children's Hospital, Boston, MA 02115; [c]Department of Genetics, Harvard Medical School, Boston, MA 02115. [d]Duke Human Vaccine Institute, Duke University School of Medicine, Durham, NC 27710.[e]Department of Surgery, Duke University, Durham, NC 27710.[f]Department of Integrative Immunobiology, Duke University School of Medicine, Durham, NC 27710. [g]Departments of Epidemiology and Microbiology and Immunology, University of North Carolina at Chapel Hill, Chapel Hill, NC 27599. [h]Biomedical Pioneering Innovation Center (BIOPIC), Peking University, Beijing 100871, [i]Changping Laboratory, Beijing 102206. [j]Division of Molecular Medicine, Boston Children's Hospital, Harvard Medical School, Boston, MA 02115, USA. [k]Department of Pediatrics, Harvard Medical School, Boston, MA 02115. [l]Department of Medicine, Duke University School of Medicine, Durham, NC 27710. [m]Department of Immunology, Duke University School of Medicine, Durham, NC 27710

[1]H.B., SL., and F.W.A. share senior authorship

[2]H.B., S.L., K.O.S., J.S.H. contributed equally to this work.

[3]Present Address: Center for Infection Biology, School of Basic Medical Sciences, Tsinghua University, Beijing, China

[4] Present Address: Department of Neurobiology, Harvard Medical School, Boston, MA 02215, USA.

[5] Present Address: Brigham and Women's Hospital, Harvard Medical School, Boston, MA 02115, USA.

[6]To whom correspondence should be addressed: Email: rbaric@email.unc.edu, yunlongcao@pku.edu.cn, barton.haynes@duke.edu, Bing.Chen@childrens.harvard.edu and alt@enders.tch.harvard.edu.

**This PDF file includes:**

**Materials and Methods**

**Figures. S1 to S14**

**Table 1**

## Materials and Methods

**Recombinant Spike protein production:** Coronavirus ectodomain proteins were produced and purified as previously described (1-4) and summarized here: SARS-CoV-2 BA.4/5 HexaPro was stabilized by introduction of prolines at amino acid positions 986 and 987. A sortase A donor sequence (LPETGG) was appended after the foldon trimerization domain to enable conjugation to ferritin nanoparticles. The plasmid encoding sortase donor-tagged SARS-CoV-2 BA.4/5 HexaPro (Genscript) were transiently transfected in FreeStyle 293-F cells (Thermo Fisher) using 293Fectin (ThermoFisher). All cells were tested monthly for mycoplasma. The 3' to the sortase A tag, the construct contained an HRV 3C-cleavage site and a C-terminal twinStrepTagII-8×His tag. Six days after transfection, the cell culture was centrifuged and filtered through a 0.8-μm filter. Spike trimers were purified from filtered cell culture supernatants using StrepTactin resin (IBA) and size-exclusion chromatography on a Superose 6 column (Cytiva) in 10 mM Tris, pH 8, 500 mM NaCl, and stored frozen at -80 C.

**Recombinant ferritin nanoparticle production:** Production was done as previously described (1-4) and summarized here: *Helicobacter pylori* ferritin particles were expressed with a pentaglycine (GGGGG) sortase A acceptor sequence encoded at the N-terminus of each subunit. For affinity purification of ferritin particles, an HRV3C cleavage site was appended to the C-terminus of each ferritin subunit followed by a 6×His tag. The gene for sortase A tagged H. pylori ferritin was cloned into VRC8400 plasmid (Genscript), and 650 μg of plasmid was transiently transfected into one liter of FreeStyle 293-F cells (Thermo Fisher) using 293Fectin (ThermoFisher). Cell culture supernatant was clarified by centrifugation at 400 x g for 1 h and filtered through a 0.8-μm filter. Clarified cell culture media was applied to a HisTrap Excel column and eluted with 0.5M imidazole using gradient elution function on an Cytiva AKTA Pure. Ferritin nanoparticles were purified by size-exclusion chromatography using Superose 6 column (Cytiva) in 10 mM Tris pH8, 500 mM NaCl, and stored frozen at -80°C.

**BA.4/5 HexaPro Sortase A conjugated nanoparticle (scNP) production.** Production was done as previously described(1-4) and summarized here: Ferritin nanoparticles with the N-terminal sortase A acceptor tag were combined with SARS-CoV-2 BA.4/5 HexaPro, and sortase A enzyme in a 10,000 MWCO VivaSpin concentrator and buffer exchanged into 50 mM Tris, 150 mM NaCl, 5 mM $CaCl_2$, pH 7.5. The final concentration of each component after concentration was 120 μM SARS-CoV-2 BA.4/5 HexaPro, 120 μM of ferritin subunit, and 100 μM of sortase A. The reaction was conducted overnight at room temperature without agitation. Following incubation, conjugated particles were isolated from free ferritin or free HexaPro by size-exclusion chromatography using a Superose 6 16/60 column. To determine the

approximate amount of coronavirus spike in 100 μg of conjugate nanoparticle, western blots of reduced Spike ectodomain scNPs were performed. Approximately 51 μg of Spike ectodomain per 100 μg of total protein was observed. unconjugated or conjugated ferritin subunits were detected by western blot with mouse polyclonal antisera and quantified by a Bio-Rad ImageDoc system. On average, 90.9% of ferritin subunits were conjugated to a Spike subunit.

**Pseudotyped neutralization assays.** Spike-pseudotyped vesicular stomatitis virus (VSV) expressing the SARS-CoV-2 variant spike glycoproteins (D614G, Beta, Gamma, Delta, BA.1, BA.2, BA.3, BA.5, BQ.1.1, XBB.1.5, JN.1, KP.2, KP.3.1.1, LP.8.1.1, MC.10.1, NB.1.8.1 and XFG) was generated as previously described(5, 6). Neutralization assays were performed as previously described and summarized here (7): Neutralization assays were conducted using the Huh-7 cell line (JCRB, 0403). Serially diluted antibodies were mixed with the pseudovirus in 96-well plates, followed by incubation for 1 hour at 37°C with 5% $CO_2$. Digested Huh-7 cells were then added and incubated for an additional 24 hours at 37°C. The cells were incubated with D-Luciferin reagent (PerkinElmer, 6066769) in the dark for 2 minutes. Cell lysates were transferred to a detection plate, and luminescence was measured using a microplate spectrophotometer (PerkinElmer, HH3400). The inhibition value for each antibody concentration was calculated as $1.0 - I_{\text{Antibody}}/\overline{I_{\text{VC}}}$, where $I_{\text{Antibody}}$ is the luminescence value of the well with a specific antibody concentration, and $I_{\text{VC}}$ is the mean value of control wells without antibody on the same plate. $IC_{50}$ values were determined by fitting a three-parameter logistic regression model with the minimum fixed at zero.

The pseudovirus neutralization assay in 293T/ACE2 cells was performed as described previously (8) and summarized here: Spike-pseudotyped virus was incubated with eight serial fivefold dilutions of antibody in duplicate for 1 h at 37 °C in 96-well plates. 293T/ACE2-MF cells were detached, resuspended in growth medium, and immediately added to all wells. Virus-only wells served as virus controls, and cell-only wells served as background controls. After 71–73 h of incubation, medium was removed and cells were lysed with 1× lysis buffer (Promega), followed by addition of Bright-Glo luciferase reagent. Luminescence was measured using a GloMax Navigator luminometer (Promega). Neutralization titers were defined as the antibody concentration that reduced relative luminescence units by 50% compared with virus control wells ($IC_{50}$).

**Deep mutational scanning (DMS) for antibody escape.** DMS was performed for the 8 new antibodies described in this paper using methods previously described(6, 9, 10) and summarized here: Yeast cells displaying RBD mutants were screened using fluorescence-activated cell sorting (FACS) to identify mutants escaping the target mAb. Cells were washed twice with 0.1% PBSA buffer by centrifugation at 2500g for 3 minutes with the supernatant

discarded after each wash. After the second wash, the cell pellet was resuspended in 0.1% PBSA buffer containing the target mAb and incubated at 4°C for 30 minutes in the dark on a rotator. After primary mAb incubation, cells were centrifuged at 2500 g for 3 minutes, the supernatant was discarded, and the pellet was washed with 1 mL of 0.1% PBSA buffer. This washing step was repeated twice. A cocktail of fluorescently labeled secondary antibodies, including goat anti-human-PE (Jackson, 109-115-098), FITC-conjugated chicken anti-C-Myc (icllab, CMYC-45F), and APC-anti-HA.11 epitope tag (Biolegend, 901524), was prepared in 0.1% PBSA buffer, filtered through a 0.22 µm filter, and added to the cells. The mixture was incubated at 4°C for 30 minutes in the dark on a rotator. Cells were then centrifuged at 2500 g for 3 minutes, the supernatant was discarded, and the pellet was resuspended in 1 mL of 0.1% PBSA buffer. The suspension was filtered through a 5 mL FACS tube with a cell strainer cap (Falcon, 352235) for FACS analysis. Using a BD FACSAria™ III flow cytometer (BD Biosciences) with a 70 µm nozzle, cells were sorted at approximately 25,000 events per second. Gating was set to select $FITC^+APC^+PE^-$ cells, indicating yeast cells expressing RBD mutants that escaped the target mAb. Sorted cells (20,000 per sample) were collected into 5 mL FACS tubes (Falcon, 352054) pre-coated with 1 mL of SD-CAA medium containing 1% (w/v) BSA. After sorting, cells were centrifuged at 3000 g for 5 minutes, 800 µL of supernatant was removed, and the cells were resuspended in 800 µL of SD-CAA medium. The suspension was transferred to a 96-well plate and cultured at 30°C with shaking for 40 hours. The cultured yeast cells were subjected to plasmid extraction using a 96-well yeast plasmid extraction kit (Coolaber, PE053). The extracted plasmids were used as templates for N26 barcode amplification by PCR. PCR products were purified with 1X Ampure XP beads (Beckman Coulter, A63882) and subjected to single-end sequencing for further analysis. The raw barcode sequencing data from FACS-based antibody DMS were processed as previously described(6, 10).

**Cryo-EM analysis of BA.5 Spike in complex with AB2-122, S212 or L52:** These analyses were performed for the 8 new antibodies described in this paper using methods previously described(11-21) and summarized here:

The full-length Omicron BA.5 spike protein containing a C-terminal strep tag was produced following a protocol described previously(11-13). To prepare the antibody-spike complexes for cryo-EM studies, the purified BA.5 spike was first mixed with SP1-77 Fab at a molar ratio of 1:6, and incubated at room temperature for 30 min. S212, L52 or AB2-122 Fab was then added to the mixture at a spike-to-Fab molar ratio of 1:6, and incubated for additional 30 min. The antibody-spike complexes were further purified by gel-filtration chromatography on a Superose 6 Increase column in a buffer containing 25 mM Tris, pH7.5, 150 mM NaCl and

0.02% DDM. The fractions containing the dissociated S1 and Fabs were pooled and concentrated. The cryo-EM grids was prepared as described before(15).

Cryo-EM images were processed in cryoSPARC v.3.3.2 with the drift correction performed and contrast transfer function estimated. Motion-corrected sums with dose weighting were used for subsequent image processing. For the S212 complex, blob picking was performed for all 13,058 recorded images, yielding 15,297,448 particles in total. These particles were subjected to multiple rounds of 2D classification, resulting in 1,380,697 particles with apparent structure features and no ice contamination. Two rounds of 3D classification were then performed and produced two major classes: class 1 included one copy of S1 and each of the two antibodies, as expected, and class 2 included two copies of the same complex in class 1. Duplicate particles were removed from the two classes, which were further NU-refined to 2.8Å resolution from 247,380 particles and 3.1Å resolution from 132,147 particles, respectively. To further improve the map, global and local CTF refinements were carried out with class 1, followed by another round of refinement with a soft mask, resulting in a final map at 2.68Å resolution for model building. Similar procedures were applied to the L52 complex. Briefly, blob picking from 24,875 recorded images gave 36,057,868 particles in total. Multiple rounds of 2D classification led to 2,782655 particles. One round of 3D classification produces two major classes, class 1 and class 2, like those from the S212 sample. After removing duplicate particles, the two classes were NU-refined, giving a map at 3.33Å resolution from 437,081 particles and another one at 3.45Å resolution from 347,356 particles, respectively. For class 1, which had only one copy of S1 and two antibodies, an additional round of 3D classification was performed to obtain a class showing good density for the entire S1 subunit. After removal of bad micrographs, re-extraction of particles, and NU-refinement, a final map at 3.27Å resolution was obtained for model building.

The AB2-122 complex dataset was processed in cryoSPARC v.4.7.0. For the AB2-122 complex, a total of 11,740 movies were recorded and subjected to patch motion correction and patch CTF estimation. After manual curation based on the CTF fit resolution, 10,252 micrographs were selected. Initial models for template picking were generated by blob picking and classified to give two classes with clear features, which were subsequently used to guide extraction of 4,834,318 particles (400 pixel box size, Fourier cropped to 200 pixels). Further rounds of 2D classification yielded 252,685 selected particles that were refined by hetero-refinement using four ab initio models to lead to the best class with 80,110 particles. These particles were re-extracted without cropping (400 pixel box size) and subjected to non-uniform refinement, producing a 3.18Å map. Reference-based motion correction followed by a final non-uniform refinement of 79,684 particles led to a 3Å map, which was locally sharpened by deepEMhancer(16) to assist model building.

All resolutions were reported from the gold-standard Fourier shell correlation using the 0.143 criterion, and local resolution was determined using cryoSPARC. For model building, our G614 S trimer structure (PDB ID: 7KRQ(12)) was used as the initial template for the S1 subunit and our SP1-77 complex structure (PDB ID: 7UPX(22)) for S212 and L52 Fabs. Several rounds of manual building were performed in Coot (17), and iterative refinement was performed in Phenix (18) and ISOLDE (19). The refinement statistics are summarized in Table Sx. More details for this protocol can be found in the Supplementary Materials. For the AB2-122 complex, an AlphaFold2 model was generated in Google Colab and rigid-body fitted into the cryo-EM map using ChimeraX(20). Manual adjustments were performed in Coot, and the model was refined using real-space refinement in Phenix under geometric restraints. Model validation was conducted using Phenix and Molprobity(21).

**Cyro-EM analysis of KP.3.1.1-Spike in complex with AB2-122.** These analyses were done as previously described(23), with some specific modifications for this complex as summarized here:

For the preparation of the antibody complex we used purified KP.3.1.1 spike. Spike protein is a soluble trimer with no transmembrane region, truncated at Q1208 with T4 foldon for enhanced trimerization and 10HIS-GS-AVI-twinStrep tags. Also, it has "6P" mutations to stabilize it and "GSAS" mutations to disrupt the furin cleavage site. The spike was mixed with a 1.5-fold molar excess of Fab BD57-2704 and AB2-122, and incubated on ice for 1 min before applying for grid preparation. An aliquot of 4 μL protein sample of AB2-122 + BD57-2704 + KP.3.1.1-Spike complex at a protein concentration of 1.0 mg/mL was loaded onto a glow-discharged 300 mesh grid (Quantifoil Au R1.2/1.3). The grids were blotted with a filter paper at 4 ℃ and 100% humidity, and flash-cooled in liquid ethane using a Thermo Fisher Vitrobot Mark IV and screened using a 200 KV Talos Aectica. Cryo-EM micrographs were collected on a 300kV Thermo Fisher Titan Krios G4 electron microscope equipped with a Falcon 4 camera and a selectris X energy filter (GIF: a slit width of 10eV). The micrographs were collected at a calibrated magnification of x130,000 using the EPU software (Thermo Fisher Scientific), yielding a pixel size of 0.95 Å at object scale. In total, 6,174 micrographs were collected at an accumulated electron dose of $60e^{-}$ $Å^{-2}$ $s^{-1}$ on each micrograph that was fractionated into a stack of 40 frames with a defocus range of -1.0 μm to −2.0 μm. CTF estimation, particle-picking, extraction, 2D classification, 3D classification, homogeneous refinement and local refinement were carried out using cryoSPARC. A total 6,174 raw micrographs were then curated to remove suboptimal data, leaving 6,143 micrographs. 2,166,882 Particles were auto-picked by the blob picker and template picker program in cryoSPARC. After several rounds of 2D classification, 316,031 particles were selected from good 2D classes and were subjected to *ab-initio*

reconstruction, followed by heterogeneous refinement. Further homogeneous refinement were conducted for 196,773 particles from the best 3D classes without applying symmetry, which resulted in a 3.36 Å map for the complex protein based on the gold-standard Fourier shell correlation criterion at FSC = 0.143. Local refinement was then calculated on the density map, focused on the region where RBD binds to BD57-2704 Fab and AB2-122 Fab with a 3.45Å local density map. The model of the complex was built by fitting a structure of the complex (predicted by AlphaFold3) into the density map using UCSF Chimera X, followed by a manual model building of the complex molecules in COOT and a real space refinement in PHENIX. The model statistics are listed in Supplementary Table 1.

**Live-virus neutralization assays:** These analyses were done as previously described(24, 25) and summarized here:

Monoclonal antibodies were standardized to 1mg/mL, plated on dilution plates at a 1:20 starting dilution, and diluted 5-fold, on a 96-well plate (Corning 3799) in live-virus medium (1X MEM, Gibco 11095080 supplemented with 5% FBS, Hyclone SH30070.03HI, and 1% Penn-Strep, Gibco 10378016). At BSL3, nanoluciferase reporter viruses were diluted in live-virus medium and added in equal volume to the dilution plates. After 1 hour at 37C, 5% CO2, the virus+serum dilutions were then transferred into duplicate columns on a 96-well black bottom plate (Corning 3916), seeded one day prior with Vero C1008 cells (ATCC CRL-1586) at 2x10^4 cells, yielding a final plated virus dilution of 800 plaque-forming units (PFU) per well. Assay plates were then incubated at 37 °C, 5% CO2 for either 24 hours (D614G and Delta VOCs) or 36 hours (BA.1, BA.5, BQ1.1, XBB1.5, JN.1, and KP.3) based on previously optimized assay conditions(24). The virus growth for each plate was quantified utilizing the Promega GloMax Explorer (GM3500) and the Nano-glo Luciferase assay system (N1130). The 50% inhibitory concentration was analyzed using the normalized non-linear regression analysis via GraphPad Prism (Version 10.4.1). Non-neutralizing antibodies were assigned a value of 2x the assay limit of detection.

**Alanine (Ala) Mutagenesis of HC-CDR3 Amino Acids and ELISA Analysis:** Ala substitution of the two adjacent aromatic residues in the HC-CDR3 of AB2-122, S212, S77, and L44 antibodies was performed using the Q5 Site-Directed Mutagenesis Kit (NEB) following the manufacturer's protocol. Antibodies were expressed in Expi293F cells (Thermo Fisher) and purified from filtered supernatants by Protein A chromatography (Cytiva). Purified IgGs were buffer exchanged into PBS. For ELISA, 96-well plates were coated overnight at 4 °C with SARS-CoV-2 Omicron JN.1 spike protein (2 μg/mL) in PBS, blocked with 1% BSA, and incubated with serial dilutions of purified antibodies for 1 hour at room temperature. Bound IgG was detected using HRP-conjugated anti-human Fc antibody.

**Fig. S1. Development of the $V_H$1-2/ hD3-3-h$J_H$6/Vκ1-33 rearranging mouse model and the characteristics of elicited antibodies.**

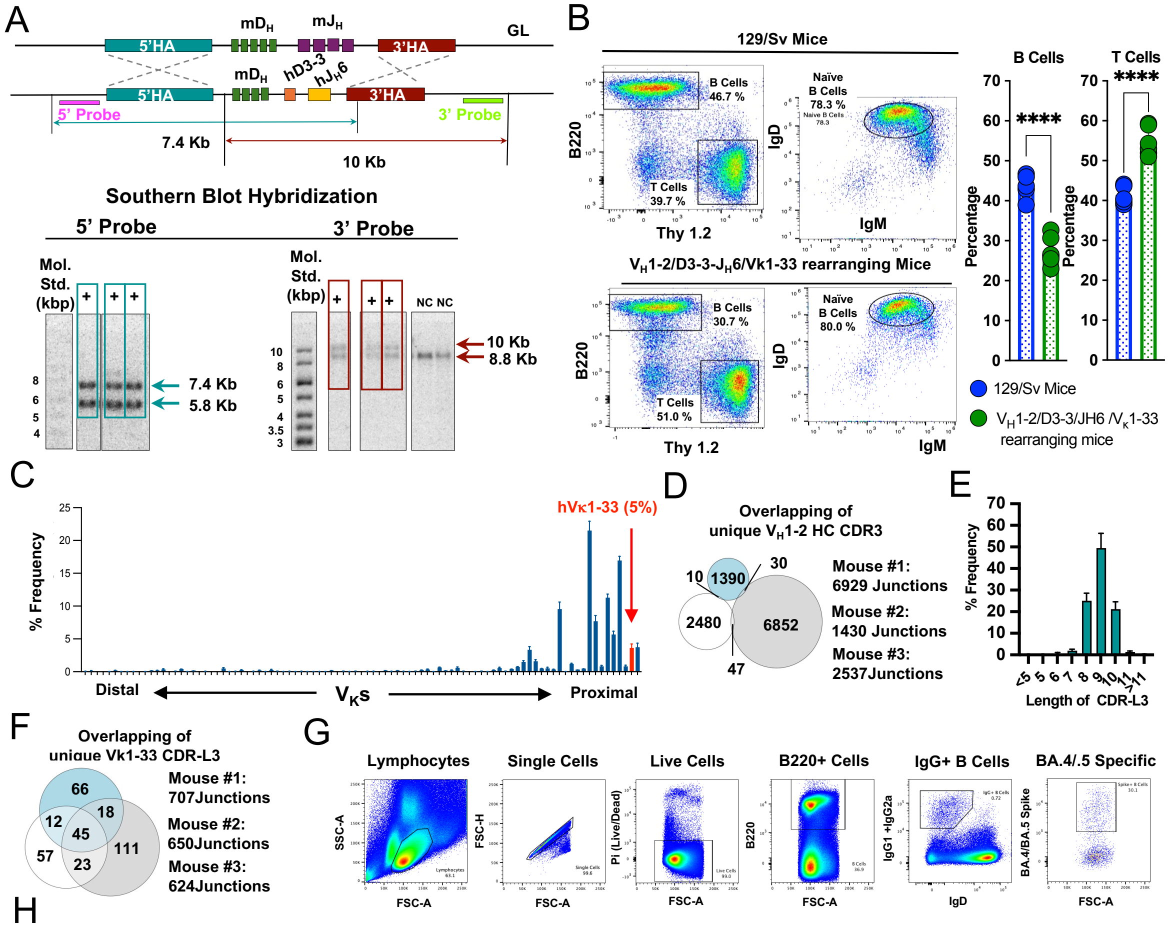


H

| mAb | B cells in lineage | Human $V_H$ | Human $J_H$ | HC CDR3 | Length of HC CDR3 | $V_H$-SHM (Amino Acid) | Human $V_K$ | Mouse $J_K$ | LC CDR3 | Length of LC CDR3 | $V_K$-SHM (Amino Acid) |
|---|---|---|---|---|---|---|---|---|---|---|---|
| AB2-05 | 4 | h$V_H$1-2 | h$J_H$6 | ARVQWLFSAMDV | 12 | 5 | hVk1-33 | mJk1 | QQYDFLSWT | 9 | 3 |
| AB2-09 | | h$V_H$1-2 | h$J_H$6 | ARVQWLFSAMDV | 12 | 4 | hVk1-33 | mJk1 | QQYDFLSWT | 9 | 3 |
| AB2-34 | | h$V_H$1-2 | h$J_H$6 | ARVQWLFSAMDV | 12 | 8 | hVk1-33 | mJk1 | QQYDFLSWT | 9 | 3 |
| AB2-60 | | h$V_H$1-2 | h$J_H$6 | ARVQWLFSAMDV | 12 | 10 | hVk1-33 | mJk1 | QQYDFLSWT | 9 | 3 |
| AB2-101 | 2 | h$V_H$1-2 | h$J_H$6 | ARRDFFNGYFDV | 12 | 17 | hVk1-33 | mJk1 | QHYDHLTWT | 9 | 5 |
| AB2-105 | | h$V_H$1-2 | h$J_H$6 | ARRDFFNGYFDV | 12 | 14 | hVk1-33 | mJk1 | QHYDHLTWT | 9 | 6 |
| AB2-113 | 2 | h$V_H$1-2 | h$J_H$6 | GRRDFWSHYMDV | 12 | 6 | hVk1-33 | mJk1 | QQYDYLSWT | 9 | 6 |
| AB2-122 | | h$V_H$1-2 | h$J_H$6 | GRRDFWSHYMDV | 12 | 7 | hVk1-33 | mJk1 | QQYDYLSWT | 9 | 5 |
| AB2-147 | 2 | h$V_H$1-2 | h$J_H$6 | ARRDFWNGYMDV | 12 | 9 | hVk1-33 | mJk1 | QHYDHLLWT | 9 | 3 |
| AB2-162 | | h$V_H$1-2 | h$J_H$6 | ARRDFWNGYMDV | 12 | 9 | hVk1-33 | mJk1 | QHYDHLLWT | 9 | 5 |

I

Pseudotyped virus Neutralization of monoclonal antibodies ($ID_{50}$: ug/ml) 293T/ACE2 cells

| | SARS-CoV-2 | | | | Omicron SARS-CoV-2 | | | | | | | | |
|---|---|---|---|---|---|---|---|---|---|---|---|---|---|
| | WT D614G | Beta | Gamma | Delta | BA.1 | BA.2 | BA.4/BA.5 | BQ.1.1 | BF.7 | XBB.1.5 | JN.1 | KP.2 | KP.3.1.1 |
| AB2-122 | >50 | >50 | >50 | >50 | 0.58 | 0.18 | 0.13 | 0.17 | 0.13 | 0.11 | 0.04 | 0.04 | 0.10 |
| | >50 | >50 | >50 | >50 | 0.76 | 0.15 | 0.09 | 0.23 | 0.17 | 0.18 | 0.08 | 0.07 | 0.19 |
| AB2-162 | >50 | >50 | >50 | >50 | 1.8 | 0.29 | 0.20 | 0.27 | 0.20 | 0.16 | 0.11 | 0.10 | 0.18 |
| | >50 | >50 | >50 | >50 | 2.1 | 0.15 | 0.16 | 0.38 | 0.27 | 0.30 | 0.14 | 0.10 | 0.41 |
| AB2-60 | >50 | >50 | >50 | >50 | 5.0 | 0.37 | 0.17 | 0.35 | 0.20 | 0.16 | 0.06 | 0.10 | 0.19 |
| | >50 | >50 | >50 | >50 | 6.4 | 0.34 | 0.15 | 0.51 | 0.32 | 0.28 | 0.20 | 0.10 | 0.28 |
| AB2-101 | >11 | >11 | >11 | >11 | 0.94 | 0.34 | 0.16 | 0.25 | 0.26 | 0.20 | 0.07 | 0.12 | 0.15 |
| | >11 | >11 | >11 | >11 | 1.0 | 0.27 | 0.21 | 0.31 | 0.23 | 0.22 | 0.19 | 0.11 | 0.40 |

Neutralization Titer; $ID_{50}$ (ug/ml)

- ≤ 0.1
- >0.1 to ≤ 0.5
- >0.5 to ≤ 1.0
- >1.0 to ≤ 5
- \> 5

**Fig. S1. Development of the $V_H$1-2/ hD3-3-h$J_H$6/Vκ1-33 rearranging mouse model and characterization of elicited antibodies. (*A*)** Top panel: Schematic illustrating the restriction endonuclease sites and Southern blot probes used to distinguish the germline configuration of the loci from the engineered *loci* in which DQ52 and mouse $J_H$1-4 were replaced with the human D3-3-$J_H$6 rearranging cassette. *Bottom panels*: Southern analysis of positive clones having hD3-3-h$J_H$6 rearranging cassette with 5' probe (pink) and 3' probe (light green). **(*B*)** (left) Representative flow cytometric plot of splenic B and T cells from 129/Sv and $V_H$1-2/ hD3-3-h$J_H$6/Vκ1-33-rearranging mice. *"(B)* (left) Representative flow cytometric plot of splenic B and T cells from 129/Sv and $V_H$1-2/ hD3-3-h$J_H$6/Vk1-33-rearranging mice. (right) Quantification of total B and T cells. Each data point represents an independent mouse; bars indicate mean ± SD. Statistical significance was determined by using an unpaired two-tailed t-test with Welch's correction. **(*C*)** Analysis of Vκ usage in $V_H$1-2/hD3-3-h$J_H$6/Vκ1-33-rearranging mouse splenic B cells as determined by HTGTS-rep-seq. The x-axis indicates all functional Vκ from the distal end of the Vκ locus to the Jκ-proximal end of the locus. The Y axis shows the percentage usage of each Vκ among all productive VκJκ rearrangements, and indicates to biased utilization of downstream Vκ in this Cer/Sis-deleted allele with that of Vκ-1-33 indicated by red arrow. **(*D*)** Venn diagrams show the $V_H$1-2 HC-CDR3 complexity. The unique reads from each library are shown to the right. The minimal overlap of $V_H$1-2 HC-CDR3 sequences across three independent mice in both models highlights the extensive CDR3 diversity**. (*E*)** Distribution of Vκ1-33 LC-CDR3 lengths in splenic B cells. Values are shown as means ± SD from three independent mouse-derived libraries. **(*F*)** Venn diagrams show the complexity of the Vκ1-33 LC-CDR3. The unique reads from each library are shown to the right. **(*G*)** FACS gating strategy used to sort single, BA.4/BA.5 SARS-CoV-2 spike-specific IgG+ B cells after immunization. **(*H*)** Table showing sequence features (V and J usage, CDR3 sequences, CDR3 length, and SHM of HC and LC variable region exons of members of the 4 selected B cell lineages. **(*I*)** The table shows the neutralization activities ($ID_{50}$ (μg/ml)) of monoclonal antibodies AB2-122, AB2-162, AB2-60, and AB2-101 performed in 293/ACE2 cells $ID_{50}$ values are also represented using the color scale shown on the right. For additional details, see the material and methods. Separate

neutralization analyses for these antibodies were performed on Huh-7 and shown in Fig.1F, using the same color scale. Neutralization values for those antibodies tested in both sets of assays are generally quite comparable. See Fig. 1 in the main text for additional details.

**Fig. S2. Complex preparation and Cryo-EM analysis of AB2-122 and SP1-77 Fabs in complex with the Omicron BA.5 S1 monomer.**

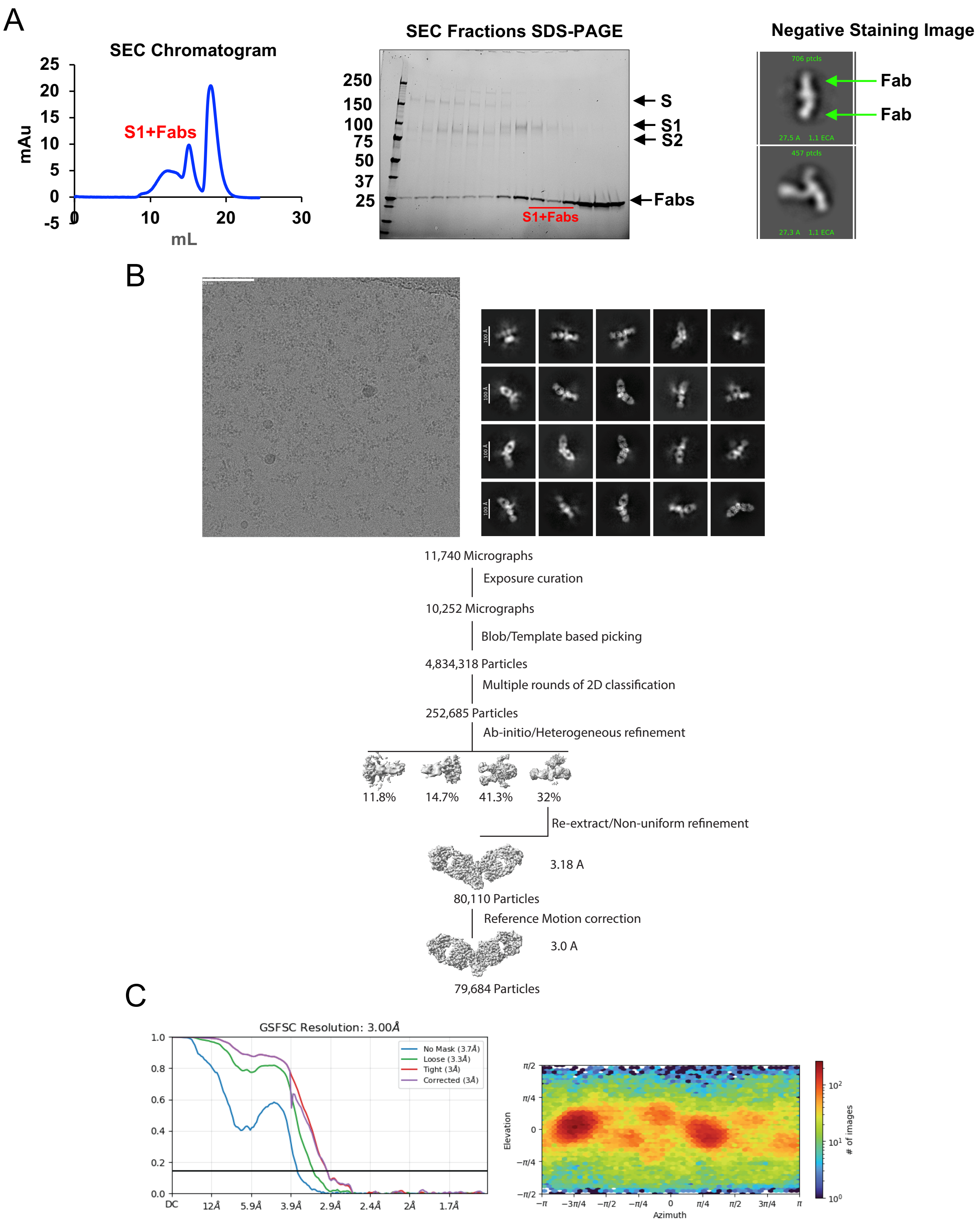

**Fig. S2. Complex preparation and Cryo-EM analysis of AB2-122 and SP1-77 Fabs in complex with the Omicron BA.5 S1 monomer. (A)** Size-exclusion chromatography (left) and SDS–PAGE analysis (middle) show the formation of S1-Fab complexes after incubation with SP1-77 plus AB2-122. Right. Negative-stain EM 2D class averages of BA.5 spike incubated with SP1-77 and AB2-122. Fab binding promotes shedding of S1, with Fabs bound to the released S1 fragments clearly visible (green arrows). Together, these biochemical and structural data demonstrate that antibody binding causes S1 dissociation from the spike.

(**B**) Left, representative motion-corrected micrograph of the vitrified complex purified by gel-filtration chromatography. Right, 2D class averages of the cryo-EM images of the Omicron BA.5 S1 monomer in complex with AB2-122 and SP1-77 Fabs from cryoSPARC; Lower, Data processing workflow for structure determination (**C**) Cryo-EM structure validation of the Fabs in complex with the Omicron BA.5 S1 monomer. FSC curves and the viewing direction distribution plot for the S1 monomer-Fabs complex.

**Fig. S3. HC-CDR3 aromatic residues are critical for targeting a mutation-exposed hydrophobic patch**

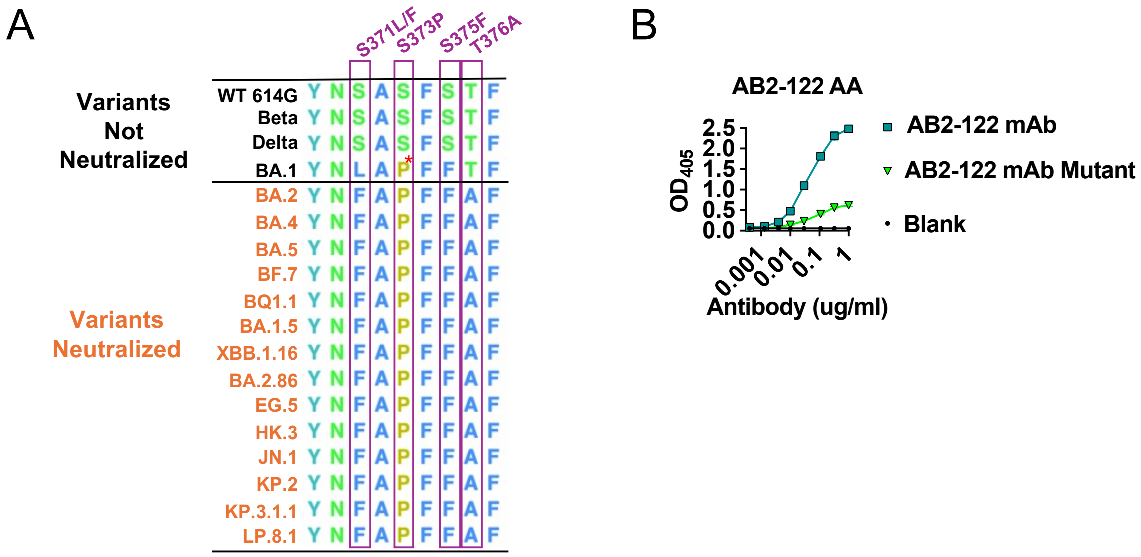

**Fig. S3. HC-CDR3 aromatic residues are critical for targeting a mutation-exposed hydrophobic patch.** (***A***) Spike protein sequence alignment showing conserved S373P (red asterisk) and putative helix-stabilizing mutations that cause an inward shift of the helix. (***B***) The two adjacent aromatic residues in the HC-CDR3 of AB2-122 were both substituted with alanines, and mutant antibodies expressed in Expi293F cells and purified by Protein A chromatography. Binding to SARS-CoV-2 Omicron JN.1 spike protein was assessed by ELISA. The X-axis shows serial antibody dilutions, and the Y-axis shows $OD_{405}$. Alanine substitution of the two adjacent aromatic residues in HC-CDR3 of AB2-122 significantly reduces binding in ELISA, confirming their critical role in epitope binding. $OD_{405}$, optical density at 405 nm. For additional details, see the supplementary material and methods. See Fig. 2 in the main text for additional details.

**Fig. S4. AB2-122 targets the KP.3.1.1 RBD epitope via two adjacent aromatic amino acid residues in its HC-CDR3**

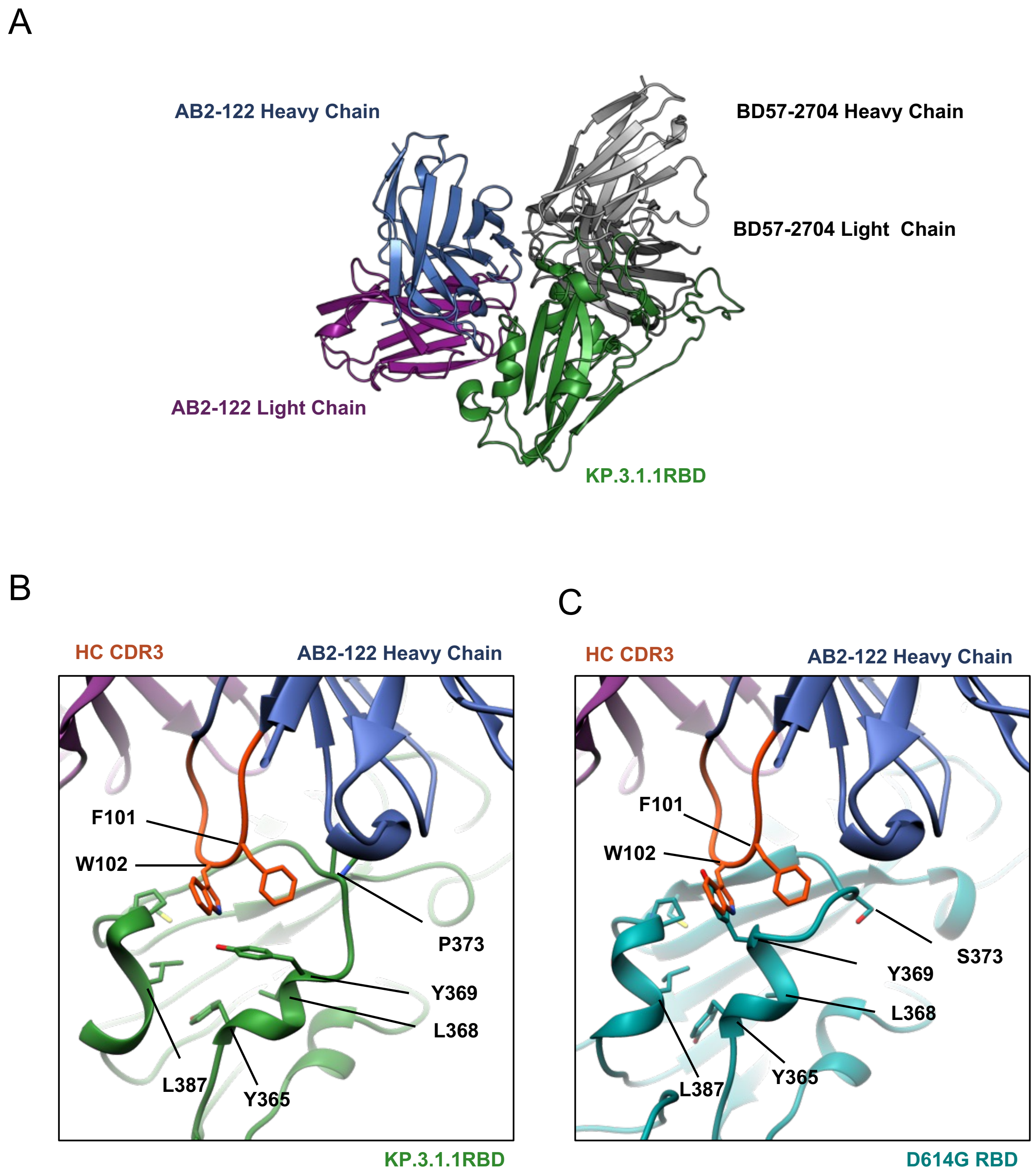

**Fig. S4. AB2-122 targets the RBD epitope via two adjacent aromatic amino acid residues in its HC-CDR3**. (***A***) Cryo-EM structure of the Omicron KP.3.1.1 S1 monomer in complex with AB2-122 and BD57-2704 Fabs in ribbon diagram, with the RBD in green, AB2-122 heavy and light chains in blue and magenta, and BD57-2704 heavy and light chains are in dark and light grey, respectively. (***B***) Close-up view of the interaction between AB2-122 Fab and the KP.3.1.1 RBD. The S212 HC-CDR3 is highlighted in orange, with Phe101 and Phe102 in stick model latched onto the hydrophobic patch on the KP.3.1.1 RBD. The contacting residues of the KP.3.1 RBD, including Y365, L368, Y369, P373 and L387, are also shown in stick model. The S373P mutation in BA.5 reconfigures the helix$^{364–372}$, exposing a hydrophobic patch that enables the two aromatic residues of AB2-122 in the HC-CDR3 to bind. (***C***) Close-up view of the modeled interaction between AB2-122 Fab and the D614G RBD (in cyan). In D614G, the unshifted helix$^{364–372}$ of the RBD clashes with the AB2-122 HC-CDR3. See Fig. 3 in the main text for additional details.

**Fig. S5. AB2-122 neutralizes SARS-CoV-2 omicron variants by blocking ACE-2 receptor binding to RBD.**

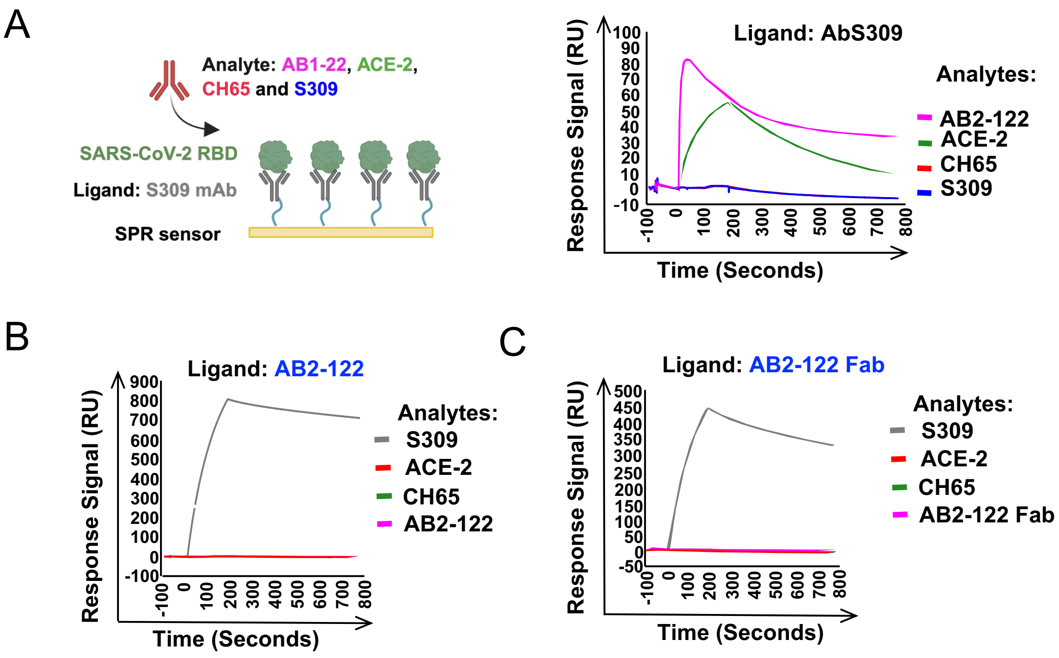


D

**Pseudotyped virus Neutralization of IgG and Fab format ($ID_{50}$: ug/ml)**

| | Omicron SARS-CoV-2 | | |
|---|---|---|---|
| | XBB.1.5 | EG.5 | JN.1 |
| | $ID_{50}$ | $ID_{50}$ | $ID_{50}$ |
| AB2-122 | 0.28 | 0.32 | 0.09 |
| AB2-122-Fab | 0.23 | 0.38 | 0.07 |

Neutralization Titer

$ID_{50}$ (ug/ml)

≤ 0.1

>0.1 to ≤ 0.5

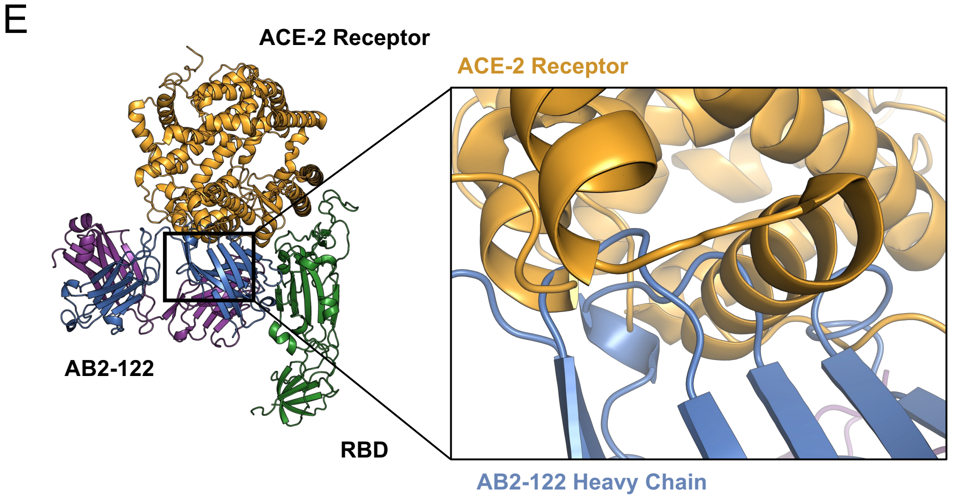

**Fig. S5. AB2-122 neutralizes SARS-CoV-2 omicron variants by blocking ACE-2 receptor binding to RBD. (A)** left. SPR competition assay schematic. Right AB2-122,ACE2-2, CH65 and S309 binding to RBD prebound to S309. **(*B*)** ACE2, S309, and CH65 binding to RBD pre-bound with AB2-122 IgG. **(*C*)** ACE2, S309, and CH65 binding to RBD pre-bound with AB2-122 Fab. S309 and CH65 are positive and negative controls, respectively. **(*D*)** Neutralization of AB2-122(IgG and Fab formats) against XBB.1.5, EG.5, and JN.1 Omicron subvariants in pseudovirus assays using 293T/ACE2 cells. ID50 values are color-coded based on the key shown at the right. Data represent two biological replicates, each with two technical replicates. **(*E*)** Potential clashes between Omicron bNAbs and ACE2 when bound to RBD. *Left*: Superposition of the structures of AB2-122 Fab shown in various colors bound to the Omicron BA.5 RBD in green, with that of the RBD in complex with ACE2 receptor in orange, reveals potential clashes between the AB2-122 and ACE2 when bound to the RBD. *Right*: Close-up view of the clashes.

## Fig. S6. BA.4/BA.5 spike-ferritin nanoparticle immunization elicits Omicron bNAbs in $V_H$1-2/Vκ1-33 mice

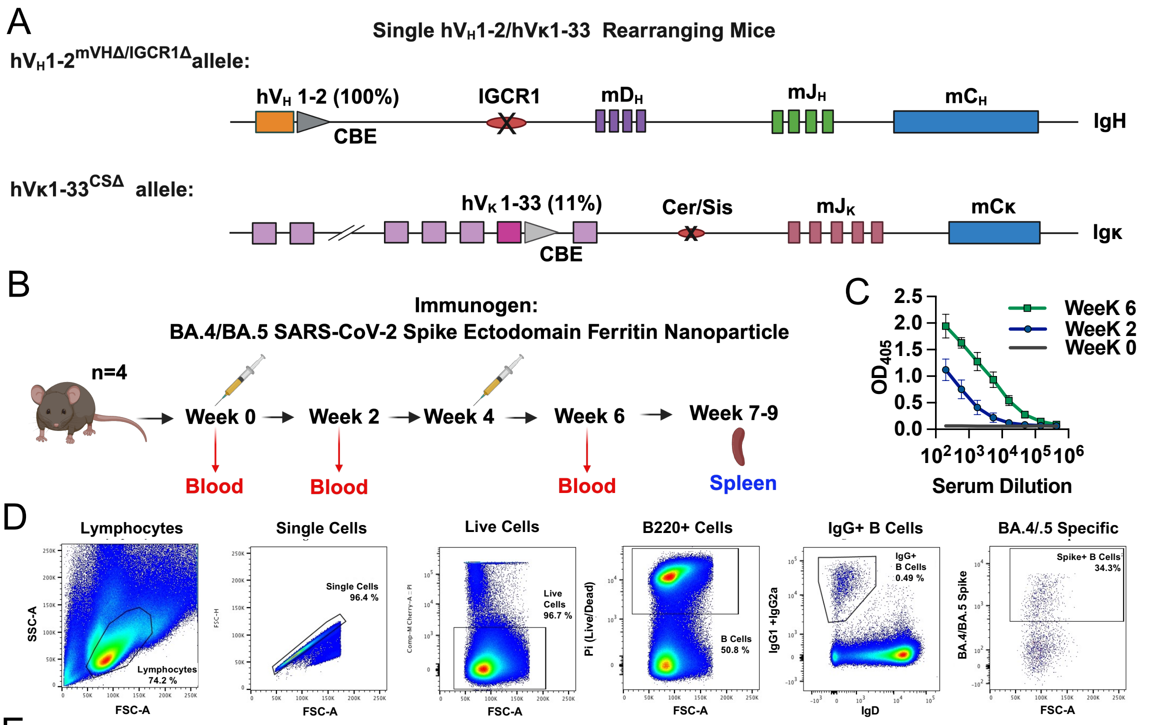


E

| mAb | B cells in lineage | Human $V_H$ | Mouse $J_H$ | HC CDR3 | Length of HC CDR3 | $V_H$-SHM (Amino Acid) | Human $V_K$ | Mouse $J_K$ | LC CDR3 | Length of LC CDR3 | $V_K$-SHM (Amino Acid) |
|---|---|---|---|---|---|---|---|---|---|---|---|
| S185 | 4 | hV$_H$1-2 | mJ$_H$1 | ARRNFFGGYFDV | 12 | 7 | hV$_K$1-33 | mJ$_K$1 | QHYDHLLWT | 9 | 5 |
| S193 | | hV$_H$1-2 | mJ$_H$1 | ARRNFFGGYFDV | 12 | 14 | hV$_K$1-33 | mJ$_K$1 | QHYDHLLWT | 9 | 5 |
| S196 | | hV$_H$1-2 | mJ$_H$1 | ARRNFFGGYFDV | 12 | 8 | hV$_K$1-33 | mJ$_K$1 | QYYDHLLWT | 9 | 6 |
| S212 | | hV$_H$1-2 | mJ$_H$1 | ARRNFFGGYFDV | 12 | 12 | hV$_K$1-33 | mJ$_K$1 | QHYDHLFWT | 9 | 5 |
| L136 | 3 | hV$_H$1-2 | mJ$_H$2 | ARRNLFYGSFDY | 12 | 14 | hV$_K$1-33 | mJ$_K$1 | QHYDHLTWT | 9 | 5 |
| L145 | | hV$_H$1-2 | mJ$_H$2 | ARRNLFYGSFDY | 12 | 16 | hV$_K$1-33 | mJ$_K$1 | QHYDHLTWT | 9 | 2 |
| L160 | | hV$_H$1-2 | mJ$_H$2 | ARRNLFYGSFDY | 12 | 17 | hV$_K$1-33 | mJ$_K$1 | QHYDHLTWT | 9 | 4 |
| L44 | 3 | hV$_H$1-2 | mJ$_H$2 | ARRDFFGGFFDF | 12 | 8 | hV$_K$1-33 | mJ$_K$1 | QHYDHLKWT | 9 | 8 |
| L57 | | hV$_H$1-2 | mJ$_H$2 | ARRDFFGGFFDF | 12 | 10 | hV$_K$1-33 | mJ$_K$1 | QHYDHLKWT | 9 | 5 |
| L61 | | hV$_H$1-2 | mJ$_H$2 | ARRDFFGGFFDF | 12 | 9 | hV$_K$1-33 | mJ$_K$1 | QHYDHLKWT | 9 | 6 |
| S77 | 2 | hV$_H$1-2 | mJ$_H$1 | ARRDYYNGYFDV | 12 | 7 | hV$_K$1-33 | mJ$_K$1 | QQYDHLSWT | 9 | 6 |
| S73 | | hV$_H$1-2 | mJ$_H$1 | ARRDYYNGYFDV | 12 | 5 | hV$_K$1-33 | mJ$_K$1 | QQYDHLSWT | 9 | 4 |

F

Pseudotyped virus Neutralization of monoclonal antibodies ($ID_{50}$: ug/ml) in Huh-7 Cells and 293/ACE2 cells

| | | SARS-CoV-2 | | | | Omicron SARS-CoV-2 | | | | | | | | | | | | | | |
|---|---|---|---|---|---|---|---|---|---|---|---|---|---|---|---|---|---|---|---|---|
| | | D614G | Beta | Gamma | Delta | BA.1 | BA.2 | BA.5 | BF.7 | BQ.1.1 | XBB.1.5 | XBB.1.16 | BA.2.86 I670V | EG.5 | HK.3 | JN.1 | KP.2 | KP.3.1.1 | LP.8.1.1 | MC.10.1 |
| Huh-7 Cells | S212 | >10 | >10 | >10 | >10 | 6.39 | 0.49 | 0.39 | N.T | 0.36 | 0.33 | N.T | N.T | N.T | N.T | 0.15 | 0.22 | 0.41 | 0.21 | 2.60 |
| | S77 | >10 | >10 | >10 | >10 | 4.21 | 0.52 | 0.38 | N.T | 0.29 | 0.63 | N.T | N.T | N.T | N.T | 0.21 | 0.14 | 0.37 | 0.16 | 2.24 |
| | L44 | >10 | >10 | >10 | >10 | 4.00 | 1.28 | 0.67 | N.T | 0.65 | 0.62 | N.T | N.T | N.T | N.T | 0.39 | 0.41 | 0.60 | 0.39 | 4.69 |
| | L52 | >10 | >10 | >10 | >10 | >10 | 0.39 | 0.29 | N.T | 0.22 | 0.63 | N.T | N.T | N.T | N.T | 0.12 | 0.13 | 0.29 | 0.12 | 2.34 |
| 293/ACE2 cells | S212 | >25 | >25 | >25 | >25 | 10 | 0.57 | 0.51 | 0.62 | 0.58 | 0.33 | 0.40 | 0.34 | 0.51 | 0.56 | 0.29 | N.T | N.T | N.T | N.T |
| | S77 | >25 | >25 | >25 | >25 | 6 | 0.49 | 0.39 | 0.48 | 0.45 | 0.37 | 0.47 | 0.30 | 0.72 | 1.1 | 0.26 | N.T | N.T | N.T | N.T |
| | L44 | >25 | >25 | >25 | >25 | 6 | 1.1 | 0.62 | 0.69 | 0.79 | 0.45 | 0.68 | 0.51 | 0.86 | 1.1 | 0.44 | N.T | N.T | N.T | N.T |
| | L52 | >25 | >25 | >25 | >25 | >25 | 0.43 | 0.43 | 0.58 | 0.51 | 0.51 | 0.51 | 0.21 | 0.66 | 1.1 | 0.28 | N.T | N.T | N.T | N.T |

Neutralization Titer

$ID_{50}$ (ug/ml)

- 0.1 to ≤ 0.5
- >0.5 to ≤ 1.0
- >1.0 to ≤ 5
- > 5
- Not Tested

**Fig. S6. BA.4/BA.5 spike-ferritin nanoparticle immunization elicits Omicron bNAbs in $V_H$1-2/Vκ1-33 mice** **(A)** Schematic illustration of *Igh* and *Igκ* loci in $V_H$1-2/Vκ1-33–rearranging mice(22). **(*B*)** Immunization scheme. Mice received a prime and a booster dose separated by 4 weeks. **(*C*)** Binding responses of sera from $V_H$1-2/Vκ1-33 mice immunized with BA.4/BA.5 spike-ferritin nanoparticles, at weeks 0, 2, and 6 against the BA.4/BA.5 spike ectodomain. Data represent mean ± SD from four mice. **(*D*)** FACS gating strategy used to sort single, BA.4/BA.5 SARS-CoV-2 spike-specific IgG+ B cells after immunization. **(*E*)** Table showing sequence features (V and J usage, CDR3 sequences, CDR3 length, and SHM of heavy and light chain) of the 4 selected B cell lineages and their members. **(*F*)** The table shows the neutralization activities (ID50(ug/ml)) of four monoclonal antibodies identified from $V_H$1-2/Vκ1-33 against SARS-CoV-2 variants in pseudovirus neutralization single assays of Huh-7cells (top) and 293/ACE2 cells (bottom). $ID_{50}$ values from the two cell-line systems are comparable, and results are displayed using the color scale shown on the right.

**Fig. S7. Single- $V_H$1-2/Vκ1-33 mice elicit Omicron bNAbs with conserved two aromatic amino acids in their 12 AA HC-CDRs3 and target the same RBD epitope.**

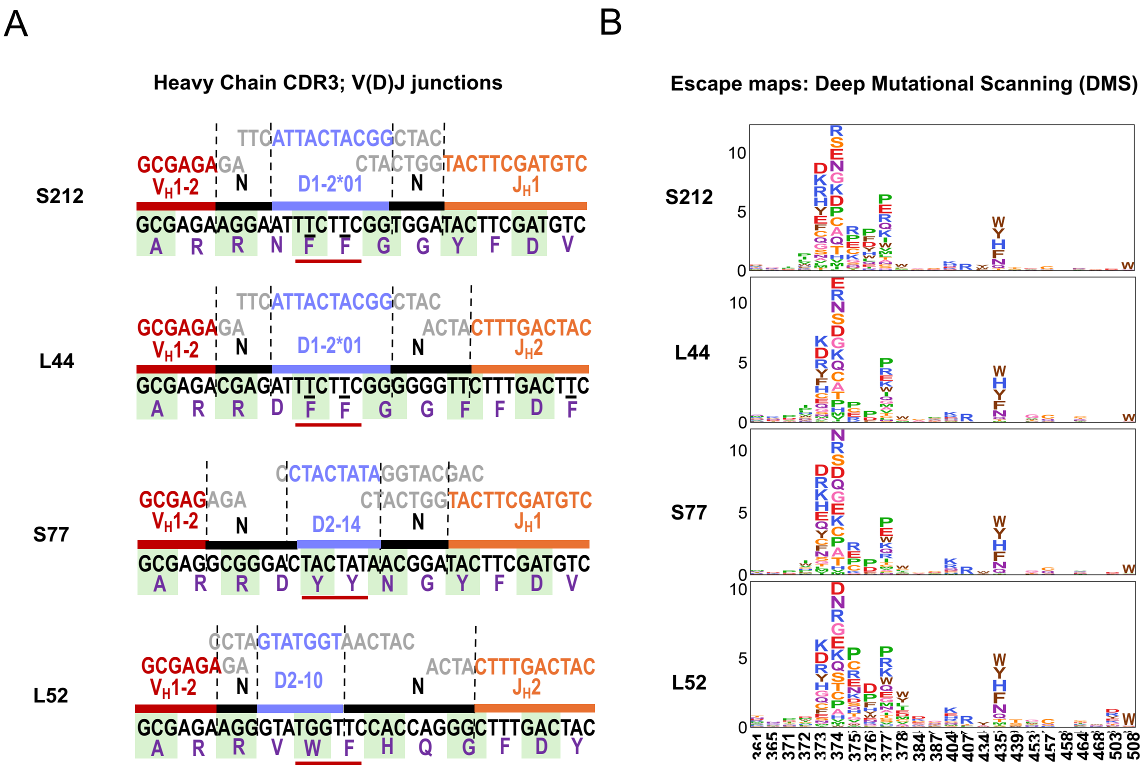

**Fig. S7. Single- $V_H$1-2/Vκ1-33 mice elicit Omicron bNAbs with conserved two aromatic amino acids in their 12 AA HC-CDRs3 and target the same RBD epitope.** (***A***) Nucleotide and amino acid sequences of HC-CDR3 V(D)J junctions from Omicron bNAbs (S212, L44, S77, and L52) isolated from $V_H$1-2/Vκ1-33 mice. The details of the presentation of this figure are the same as those in the legend to main Fig. 2. As shown in this figure, all four Omicron-neutralizing antibodies isolated from the Single-$V_H$1-2/Vκ1-33 contain two conserved aromatic residues within their HC-CDR3s. Two adjacent aromatic residues centrally located within the HC-CDR3 are highlighted (red underline) for each HC-CDR3 sequence (***B***) Deep mutational scanning (DMS) escape maps for the corresponding antibodies are shown as logo plots across the RBD. Amino acids are colored on the basis of their chemical properties, and letter height in the logo plots indicates escape scores. More details on the DMS method have been described (9). See Fig. 2 in the main text for additional details.

**Fig. S8. Complex preparation and Cryo-EM analysis of S212 and SP1-77 Fabs in complex with the Omicron BA.5 S1 monomer.**

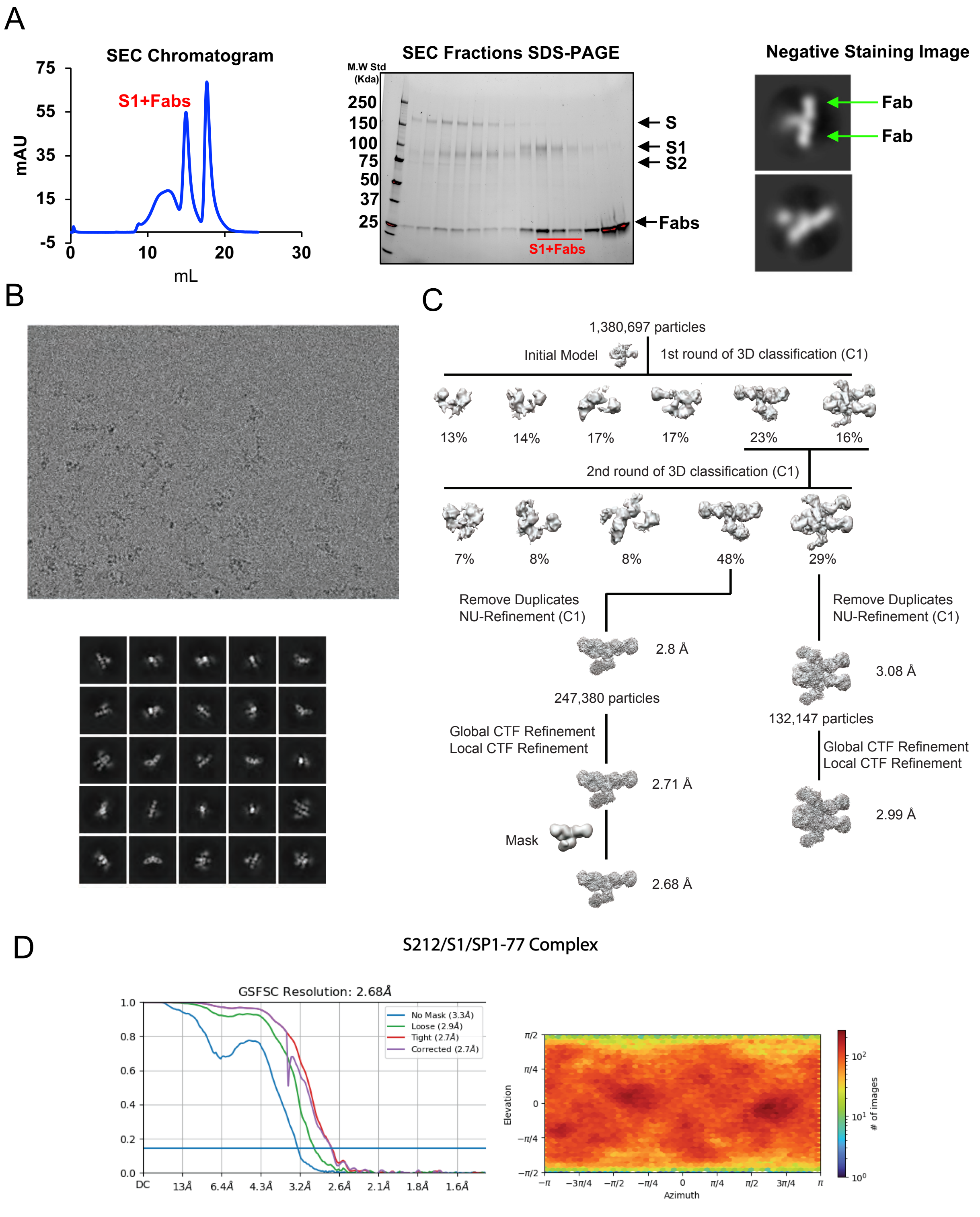

**Fig. S8. Complex preparation and Cryo-EM analysis of S212 and SP1-77 Fabs in complex with the Omicron BA.5 S1 monomer. (A)** Size-exclusion chromatography (left) and SDS–PAGE analysis (middle) show the formation of S1-Fab complexes after incubation with SP1-77 plus S212. Right. Negative-stain EM 2D class averages of BA.5 spike incubated with SP1-77 and S212. Fab binding promotes shedding of S1, with Fabs bound to the released S1 fragments clearly visible (green arrows). Together, these biochemical and structural data demonstrate that antibody binding causes S1 dissociation from the spike. (**B**) upper, representative motion-corrected micrograph of the vitrified complex purified by gel-filtration chromatography. Lower, 2D class averages of the cryo-EM images of the Omicron BA.5 S1 monomer in complex with S212 and SP1-77 Fabs from cryoSPARC; (**C**) Data processing workflow for structure determination (**D**) Cryo-EM structure validation of the Fabs in complex with the Omicron BA.5 S1 monomer. FSC curves and the viewing direction distribution plot for the S1 monomer-Fabs complex.

**Fig S9. S212 targets the RBD epitope via two adjacent aromatic amino acid residues in its HC-CDR3.**

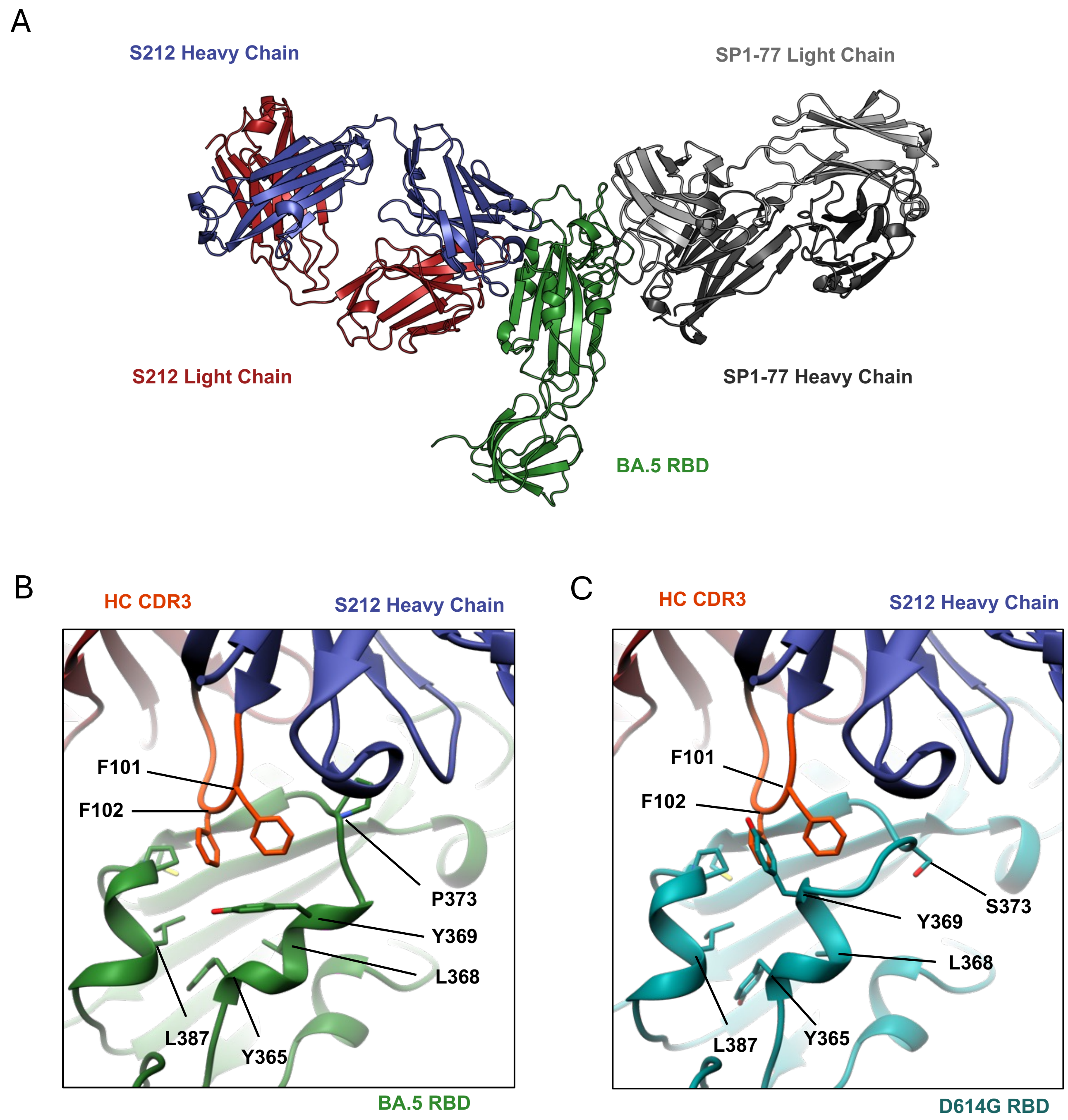

**Fig S9. S212 targets the RBD epitope via two adjacent aromatic amino acid residues in its HC-CDR3**. (***A***) Cryo-EM structure of the Omicron BA.5 S1 monomer in complex with S212 and SP1-77 Fabs in ribbon diagram, with the RBD in green, S212 heavy and light chains in blue and red, and SP1-77 heavy and light chains in grey and light grey, respectively. (***B***) Close-up view of the interaction between S212 Fab and the BA.5 RBD. The S212 HC-CDR3 is highlighted in orange, with Phe101 and Phe102 in stick model latched onto the hydrophobic patch on the BA.5 RBD. The contacting residues of the BA.5 RBD, including Y365, L368, Y369, P373 and L387, are also shown in stick model. The S373P mutation in BA.5 reconfigures the helix$^{364–372}$, exposing a hydrophobic patch that enables the two aromatic residues of S212 in the HC-CDR3 to bind. (***C***) Close-up view of the modeled interaction between S212 Fab and the D614G RBD (in cyan). In D614G, the unshifted helix$^{364–372}$ of the RBD clashes with the S212 HC-CDR3. See Fig. S8 in the for additional details.

**Fig. S10. Complex preparation and Cryo-EM analysis of L52 and SP1-77 Fabs in complex with the Omicron BA.5 S1 monomer.**

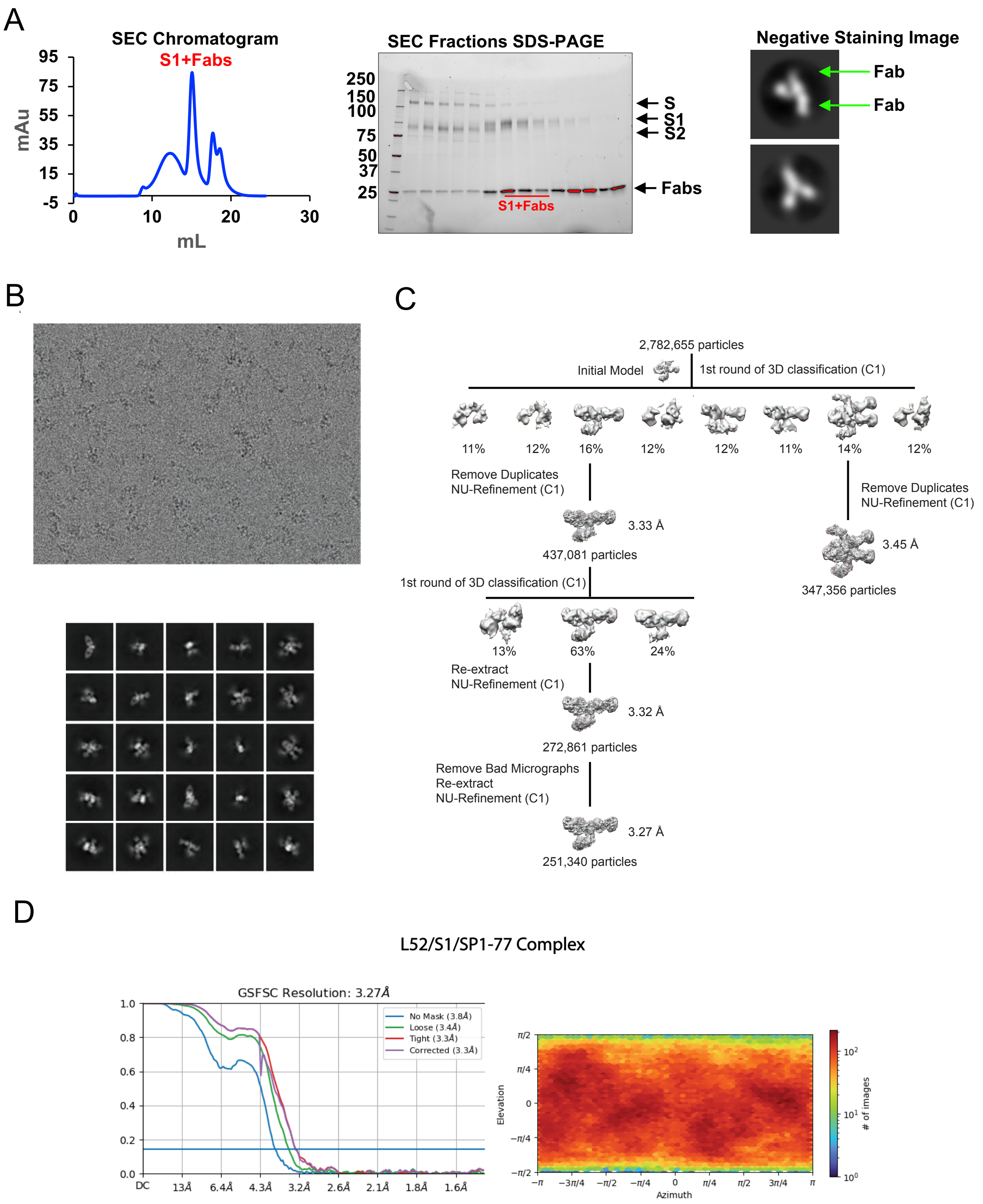

**Fig. S10. Complex preparation and Cryo-EM analysis of L52 and SP1-77 Fabs in complex with the Omicron BA.5 S1 monomer. (A)** Size-exclusion chromatography(left) and SDS–PAGE analysis (middle) show the formation of S1-Fab complexes after incubation with SP1-77 plus L52. Right. Negative-stain EM 2D class averages of BA.5 spike incubated with SP1-77 and L52. Fab binding promotes shedding of S1, with Fabs bound to the released S1 fragments clearly visible (green arrows). Together, these biochemical and structural data demonstrate that antibody binding causes S1 dissociation from the spike. (**B**) upper, representative motion-corrected micrograph of the vitrified complex purified by gel-filtration chromatography. Lower, 2D class averages of the cryo-EM images of the Omicron BA.5 S1 monomer in complex with L52 and SP1-77 Fabs from cryoSPARC; (**C**) Data processing workflow for structure determination (**D**) Cryo-EM structure validation of the Fabs in complex with the Omicron BA.5 S1 monomer. FSC curves and the viewing direction distribution plot for the S1 monomer-Fabs complex.

**Fig. S11. L52 targets the RBD epitope via two adjacent aromatic amino acid residues in its HC-CDR3.**

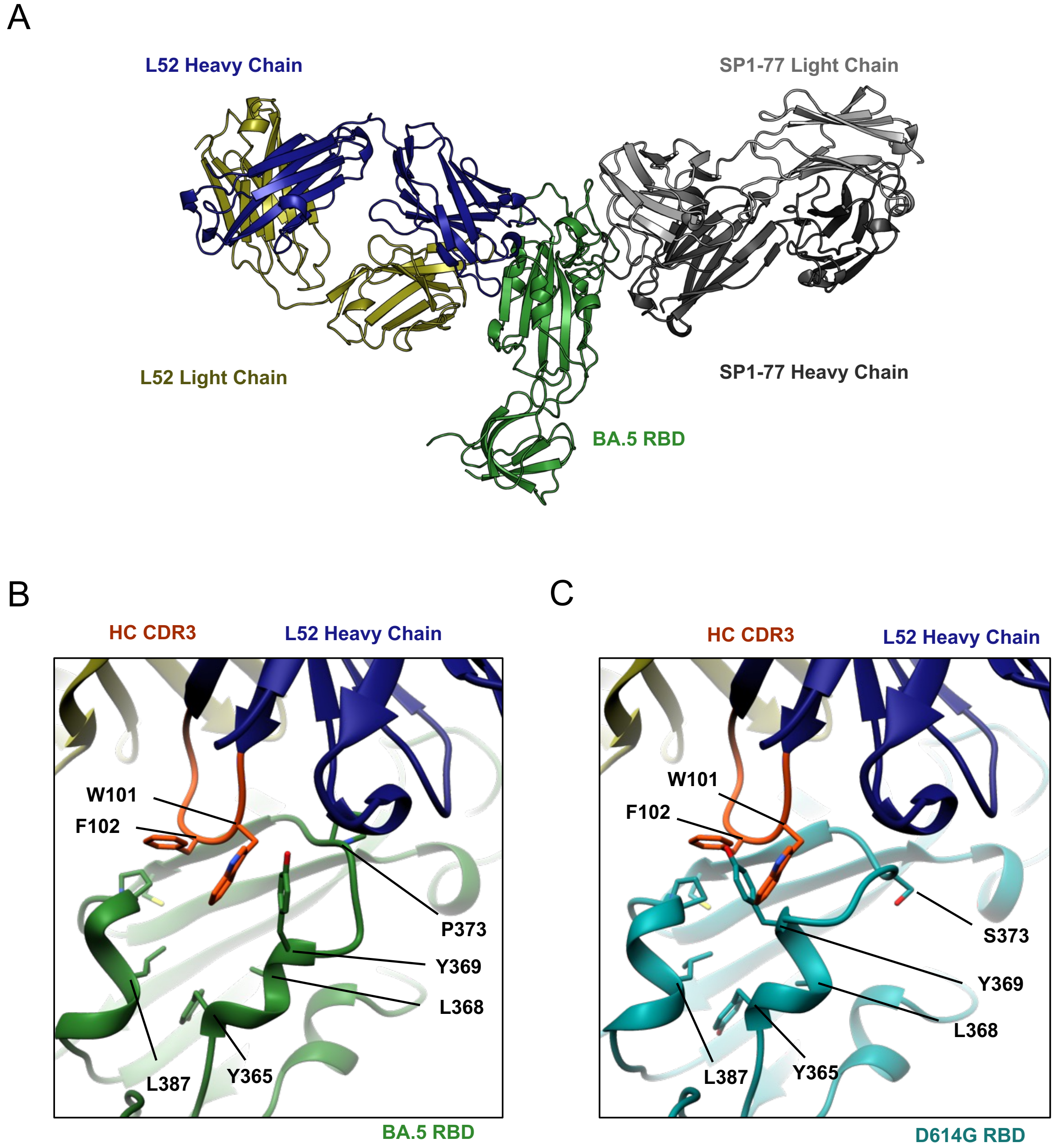

**Fig. S11. L52 targets the RBD epitope via two adjacent aromatic amino acid residues in its HC-CDR3**. (***A***) Cryo-EM structure of the Omicron BA.5 S1 monomer in complex with L52 and SP1-77 Fabs, with the RBD in green, L52 heavy and light chains in blue and olive green, and SP1-77 heavy and light chains are in grey and light grey, respectively. (***B***) Close-up view of the interaction between L52 Fab and the BA.5 RBD, with the L52 HC-CDR3 highlighted in orange, and Trp101 and Phe102 in stick model. The contacting residues of the BA.5 RBD, including Y365, L368, Y369, P373, and L387, are also shown in stick model. The S373P mutation in BA.5 reconfigures the helix$^{364–372}$, exposing a hydrophobic patch that enables the two aromatic residues of L52 in the HC-CDR3 to bind. (***C***) Close-up view of the modeled interaction between L52 Fab and the D614G RBD (in cyan). In D614G, the unshifted helix$^{364–372}$ of the RBD clashes with the L52 HC-CDR3. See Fig. S10 in the for additional details.

**Fig. S12. Two aromatic amino acids in the HC-CDR3 in omicron bNAbs from $V_H$1-2/Vκ1-33 mouse model are critical for binding JN.1 Spike.**

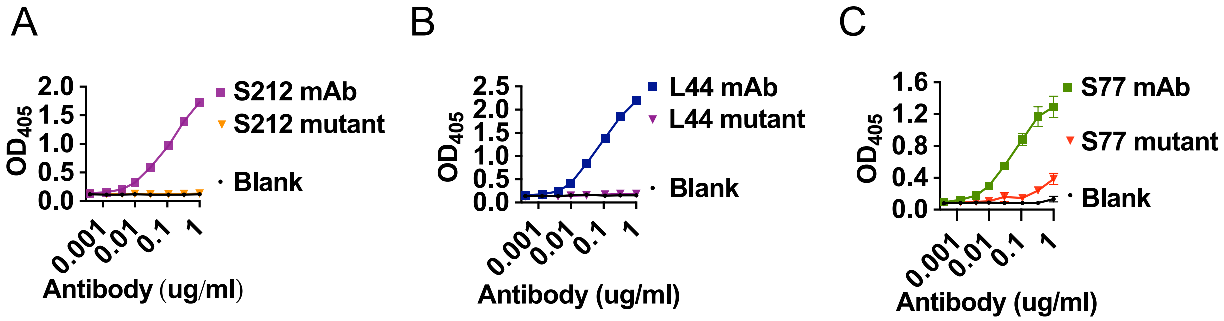

**Fig. S12. Two aromatic amino acids in the HC-CDR3 in omicron bNAbs from $V_H$1-2/Vκ1-33 mouse model are critical for binding JN.1 Spike. (*A to C*)**. The two adjacent aromatic residues in the HC-CDR3 of S212, S77, and L44 were substituted with alanines, and mutant antibodies were expressed in Expi293F cells and purified by Protein A chromatography. Binding to SARS-CoV-2 Omicron JN.1 spike protein was assessed by ELISA. The X-axis shows serial antibody dilutions, and the Y-axis shows $OD_{405}$. Alanine substitution of the two adjacent aromatic residues in HC-CDR3 of these antibodies significantly reduces binding in ELISA, confirming their critical role in epitope binding. $OD_{405}$, optical density at 405 nm. For additional details, see the supplementary material and methods. See Fig. S7 in the for additional details.

**Fig. S13. Omicron bNAbs neutralize SARS-CoV-2 by blocking ACE2 receptor binding to RBD.**

A

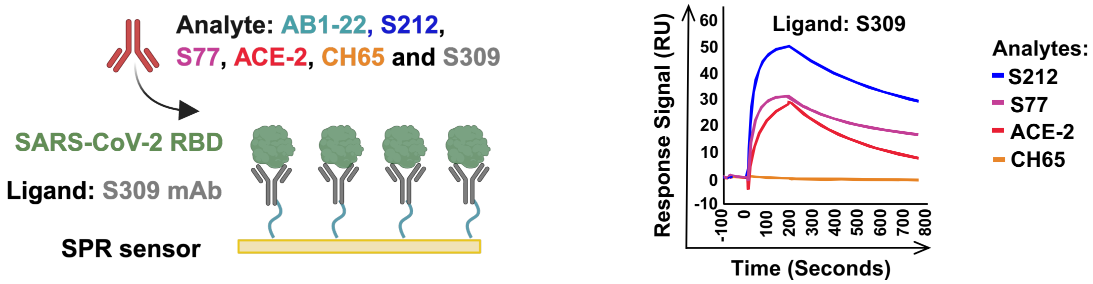


B

C

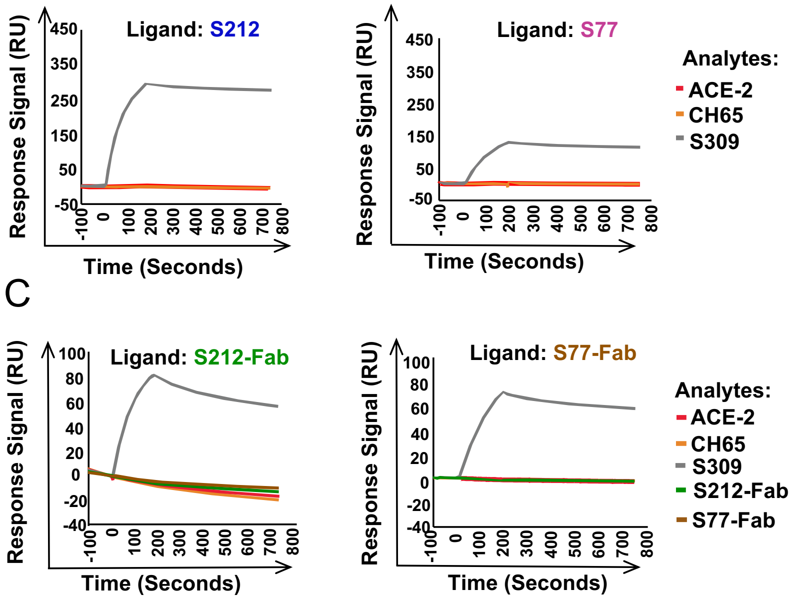


D

Pseudotyped virus Neutralization of IgG and Fab format ($ID_{50}$: ug/ml)

| | Omicron SARS-CoV-2 | | | | | |
|---|---|---|---|---|---|---|
| | XBB.1.5 | | EG.5 | | JN.1 | |
| | $ID_{50}$ | | $ID_{50}$ | | $ID_{50}$ | |
| S212 | 0.37 | 0.37 | 0.48 | 0.46 | 0.20 | 0.18 |
| S212-Fab | 0.67 | 0.79 | 0.67 | 0.79 | 0.32 | 0.35 |
| S77 | 0.37 | 0.59 | 0.46 | 0.63 | 0.15 | 0.17 |
| S77-Fab | 0.59 | 0.64 | 0.83 | 0.87 | 0.26 | 0.29 |

Neutralization Titer $ID_{50}$ (ug/ml)

- 0.1 to ≤ 0.5
- >0.5 to ≤ 1.0

**Fig. S13. Omicron bNAbs neutralize SARS-CoV-2 by blocking ACE2 receptor binding to RBD. (*A*)** SPR competition assay schematic (left) and traces (right) showing ACE2 (red), S212 (blue), S77 (magenta), S309 (grey), and CH65 (orange) binding to a pre-formed S309–RBD complex. **(*B*)** ACE2, S309, and CH65 binding to RBD pre-bound with S212 or S77 IgG. **(*C*)** Bottom panels: ACE2, S212 Fab, S77 Fab, S309, and CH65 binding to RBD pre-bound with S212 or S77 Fabs. S309 and CH65 are positive and negative controls, respectively. **(*D*)** Neutralization of S212 and S77 (IgG and Fab formats) against XBB.1.5, EG.5, and JN.1 Omicron subvariants in pseudovirus assays using 293T/ACE2 cells. ID50 values are color-coded based on the key shown at the right. Data represent two biological replicates, each with two technical replicates.

**Fig. S14. Potential clashes between Omicron bNAbs and ACE2 when bound to RBD.**

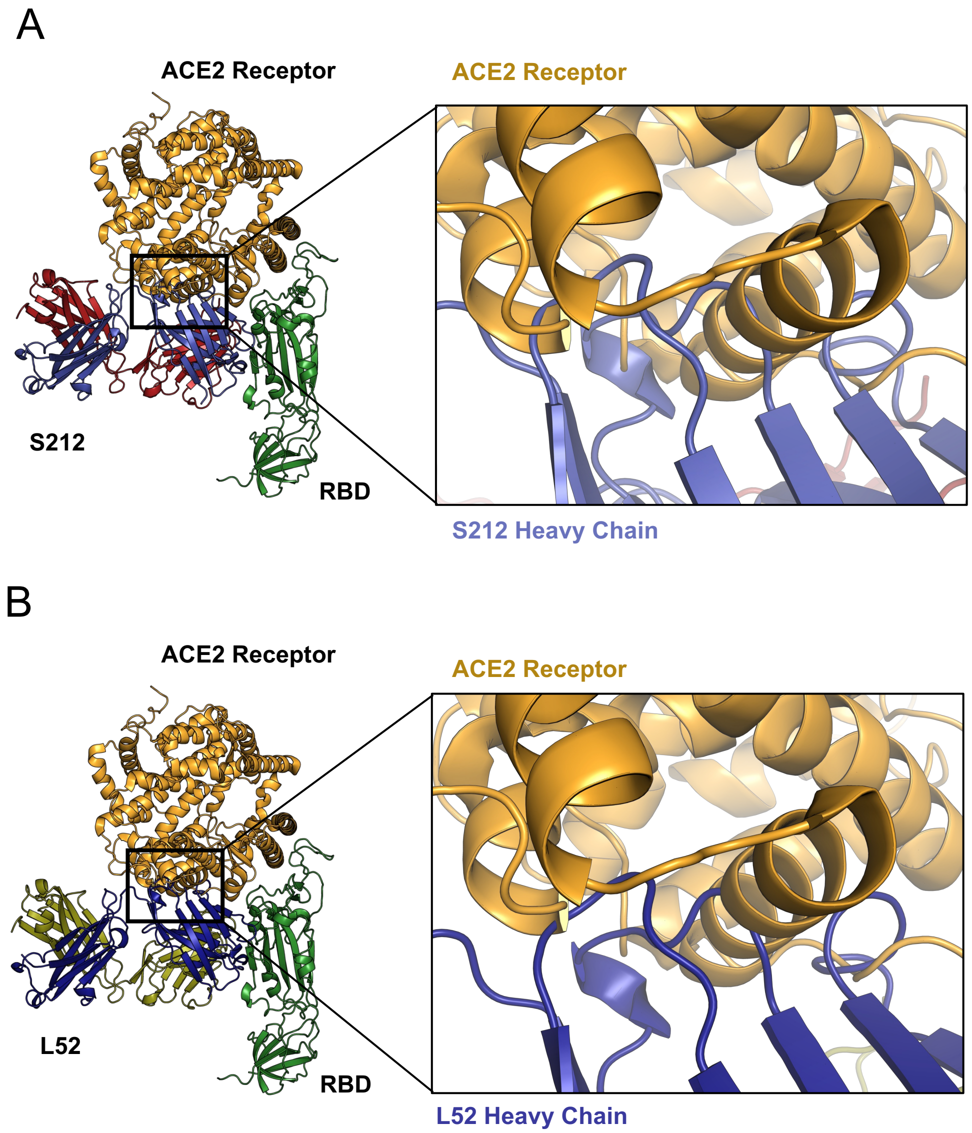

**Fig. S14. Potential clashes between Omicron bNAbs and ACE2 when bound to RBD. (*A and B*)** *Left*: Superposition of the structures of S212, and L52 Fabs shown in various colors bound to the Omicron BA.5 RBD in green, with that of the RBD in complex with ACE2 in orange reveals potential clashes between the antibodies and ACE2 when bound to the RBD. *Right*: Close-up view of the clashes.

## Table 1. Cryo-EM data collection, refinement, and validation statistics

| | L52/S1/SP1-77 (EMD-73447) (PDB 9YTA) | S212/S1/SP1-77 (EMD-73457) (PDB 9YTC) | AB2-122/S1/SP1-77 (EMD-73392) (PDB 9YSG) | AB2-122 + BD57-2704 + KP.3.1.1-Spike (EMD-66671)(PDB-9X9T) |
|---|---|---|---|---|
| **Data collection and processing** | | | | |
| Magnification | 165,000 | 165,000 | 165,000 | 130,000 |
| Voltage (kV) | 300 | 300 | 300 | 300 |
| Electron exposure (e–/Å$^2$) | 53.69 | 54.09 | 52.28 | 60 |
| Defocus range (μm) | -0.5~-2.2 | -0.5~-2.2 | -0.5~-2.2 | -1.0~-2.0 |
| Pixel size (Å) | 0.73 | 0.73 | 0.736 | 0.95 |
| Symmetry imposed | C1 | C1 | C1 | C1 |
| Initial particle images (no.) | 28,778,303 | 15,297,448 | 4,834,318 | 2,166,882 |
| Final particle images (no.) | 251,340 | 247,380 | 79,684 | 196,773 |
| Map resolution (Å) | 3.27 | 2.68 | 3.00 | 3.45 |
| FSC threshold | 0.143 | 0.143 | 0.143 | 0.143 |
| Final map sampling (Å/pix) | 0.73 | 0.73 | 0.736 | 0.95 |
| **Refinement** | | | | |
| Initial model used (PDB code) | 7KRQ,7UPX | 7KRQ,7UPX | 7KRQ,7UPX, AlphaFold2 | AlphaFold3 |
| Model resolution (Å) | 3.4 | 2.9 | 3.2 | |
| FSC threshold | 0.5 | 0.5 | 0.5 | 0.5 |
| Map sharpening *B* factor (Å$^2$) | -107.0 | -82.3 | -89.4 | -75.9 |
| Model composition | | | | |
| Protein residues | 1140 | 1140 | 1141 | 624 |
| Ligands | NAG:3, FUC:1 | NAG:3, FUC:1 | NAG:3, FUC:1 | 0 |
| *B* factors (Å$^2$) | | | | |
| Protein (min/max/mean) | 90.92/275.67/150.44 | 70.74/239.79/124.99 | 48.59/238.62/123.35 | |
| Ligand (min/max/mean) | 143.65/210.77/177.86 | 103.34/173.73/135 | 96.60/141.82/125.48 | |
| R.m.s. deviations | | | | |
| Bond lengths (Å) | 0.004 | 0.004 | 0.004 | 0.004 |
| Bond angles (°) | 0.945 | 0.978 | 0.962 | 0.577 |
| Validation | | | | |
| MolProbity score | 1.78 | 1.58 | 1.55 | |
| Clashscore | 6.33 | 4.65 | 4.03 | 9.08 |
| Poor rotamers (%) | 1.32 | 0.2 | 0.81 | 0 |
| Ramachandran plot | | | | |
| Favored (%) | 95.04 | 95.12 | 94.68 | 91.83 |
| Allowed (%) | 4.78 | 4.70 | 5.23 | 8.01 |
| Disallowed (%) | 0.18 | 0.18 | 0.09 | 0.16 |

## References


1. D. Li *et al.*, In vitro and in vivo functions of SARS-CoV-2 infection-enhancing and neutralizing antibodies. *Cell* **184**, 4203–4219 e4232 (2021).
2. K. O. Saunders *et al.*, Neutralizing antibody vaccine for pandemic and pre-emergent coronaviruses. *Nature* **594**, 553–559 (2021).
3. D. Wrapp *et al.*, Cryo-EM structure of the 2019-nCoV spike in the prefusion conformation. *Science* **367**, 1260–1263 (2020).
4. T. Zhou *et al.*, Structure-Based Design with Tag-Based Purification and In-Process Biotinylation Enable Streamlined Development of SARS-CoV-2 Spike Molecular Probes. *Cell Rep* **33**, 108322 (2020).
5. H. Li *et al.*, Establishment of replication-competent vesicular stomatitis virus-based recombinant viruses suitable for SARS-CoV-2 entry and neutralization assays. *Emerg Microbes Infect* **9**, 2269–2277 (2020).
6. A. Yisimayi *et al.*, Repeated Omicron exposures override ancestral SARS-CoV-2 immune imprinting. *Nature* **625**, 148–156 (2024).
7. J. Nie *et al.*, Establishment and validation of a pseudovirus neutralization assay for SARS-CoV-2. *Emerg Microbes Infect* **9**, 680–686 (2020).
8. P. B. Gilbert *et al.*, Immune correlates analysis of the mRNA-1273 COVID-19 vaccine efficacy clinical trial. *Science* **375**, 43–50 (2022).
9. Y. Cao *et al.*, Rational identification of potent and broad sarbecovirus-neutralizing antibody cocktails from SARS convalescents. *Cell Rep* **41**, 111845 (2022).
10. F. Jian *et al.*, Evolving antibody response to SARS-CoV-2 antigenic shift from XBB to JN.1. *Nature* **637**, 921–929 (2025).
11. Y. Cai *et al.*, Distinct conformational states of SARS-CoV-2 spike protein. *Science* **369**, 1586–1592 (2020).
12. J. Zhang *et al.*, Structural impact on SARS-CoV-2 spike protein by D614G substitution. *Science* **372**, 525–530 (2021).
13. J. Zhang *et al.*, Membrane fusion and immune evasion by the spike protein of SARS-CoV-2 Delta variant. *Science* **374**, 1353–1360 (2021).
14. A. Punjani, J. L. Rubinstein, D. J. Fleet, M. A. Brubaker, cryoSPARC: algorithms for rapid unsupervised cryo-EM structure determination. *Nat Methods* **14**, 290–296 (2017).
15. D. N. Mastronarde, Automated electron microscope tomography using robust prediction of specimen movements. *J Struct Biol* **152**, 36–51 (2005).
16. R. Sanchez-Garcia *et al.*, DeepEMhancer: a deep learning solution for cryo-EM volume post-processing. *Commun Biol* **4**, 874 (2021).
17. P. Emsley, K. Cowtan, Coot: model-building tools for molecular graphics. *Acta Crystallogr D Biol Crystallogr* **60**, 2126–2132 (2004).
18. P. D. Adams *et al.*, PHENIX: a comprehensive Python-based system for macromolecular structure solution. *Acta Crystallogr D Biol Crystallogr* **66**, 213–221 (2010).
19. T. I. Croll, ISOLDE: a physically realistic environment for model building into low-resolution electron-density maps. *Acta Crystallogr D Struct Biol* **74**, 519–530 (2018).

20. E. C. Meng *et al.*, UCSF ChimeraX: Tools for structure building and analysis. *Protein Sci* **32**, e4792 (2023).
21. C. J. Williams *et al.*, MolProbity: More and better reference data for improved all-atom structure validation. *Protein Sci* **27**, 293–315 (2018).
22. S. Luo *et al.*, An antibody from single human V(H)-rearranging mouse neutralizes all SARS-CoV-2 variants through BA.5 by inhibiting membrane fusion. *Sci Immunol* **7**, eadd5446 (2022).
23. Y. Cao *et al.*, Characterization of the enhanced infectivity and antibody evasion of Omicron BA.2.75. *Cell Host Microbe* **30**, 1527–1539 e1525 (2022).
24. W. N. Voss *et al.*, Hybrid immunity to SARS-CoV-2 arises from serological recall of IgG antibodies distinctly imprinted by infection or vaccination. *Cell Rep Med* **5**, 101668 (2024).
25. D. R. Martinez *et al.*, A broadly cross-reactive antibody neutralizes and protects against sarbecovirus challenge in mice. *Sci Transl Med* **14**, eabj7125 (2022).
26. R. Pincus, Aitchison, J.: The Statistical Analysis of Compositional Data. Chapman and Hall, London - New York 1986, XII, 416 pp., £ 25,00. *Biometrical Journal* **30**, 794–794 (1988).
